# Plant Stress Signaling in a Cross-Kingdom Context: Metabolic Control of Redox and ROS-Ca^2+^-Hormone Interactomes

**DOI:** 10.3390/plants15192972

**Published:** 2026-09-29

**Authors:** Mohamad Warda, Merve Bolat, Samet Tekin, Burak Çinar, Aslıhan Atasever, Emin Şengül, Fikret Çelebi, Ali Cinar, Mahmut Sinan Taspinar, Bushra Y. Ahmed, Ângelo de Fátima, Andreia C. S. Ferreira, Luis F. M. P. Souza, Jeong-In Baek, Luzia V. Modolo, Jaehoon Bae, A. M. Abd El-Aty

**Affiliations:** 1 Department of Physiology, Faculty of Veterinary Medicine, Atatürk University, Erzurum 25240, Turkey; mervebolat@atauni.edu.tr (M.B.); samet.tekin@atauni.edu.tr (S.T.); emin.sengul@atauni.edu.tr (E.Ş.); fncelebi@atauni.edu.tr (F.Ç.); d.cinar@atauni.edu.tr (A.C.); 2 Department of Biochemistry and Molecular Biology, Faculty of Veterinary Medicine, Cairo University, Giza 12211, Egypt; 3 Daytam East Anatolian High Technology Application and Research Center (DAYTAM), Atatürk University, Erzurum 25240, Turkey; burakcinar@atauni.edu.tr; 4 Department of Veterinary Medicine, Çayırlı Vocational School, Erzincan Binali Yıldırım University, Erzincan 24500, Turkey; aslihan.atasever@erzincan.edu.tr; 5 Department of Agricultural Biotechnology, Faculty of Agriculture, Atatürk University, Erzurum 25240, Turkey; taspinar@atauni.edu.tr; 6 Institute for Health & Wellbeing Research, University of Bedfordshire, University Square, Luton LU1 3JU, UK; bushra.ahmed1@beds.ac.uk; 7 Departamento de Química, Instituto de Ciências Exatas, Universidade Federal de Minas Gerais, Belo Horizonte 31270-901, MG, Brazil; adefatima.geqob@gmail.com; 8 Department of Organic Chemistry, Faculty of Science, Atatürk University, Erzurum 25240, Turkey; 9 Departamento de Botânica, Instituto de Ciências Biológicas, Universidade Federal de Minas Gerais, Belo Horizonte 31270-901, MG, Brazil; andreiac09@gmail.com (A.C.S.F.); luisfmps@gmail.com (L.F.M.P.S.); 10 Department of Companion Animal Health, College of K-Bio Health, Daegu Haany University, Gyeongsan 38610, Republic of Korea; baek@dhu.ac.kr; 11 Department of Food Industry, Daegu Haany University, Gyeongsan 38610, Republic of Korea; 12 Industry-University Cooperation Foundation, Functional Food Research Institute, Daegu Haany University, Gyeongsan 38610, Republic of Korea; 13 Department of Pharmacology, Faculty of Veterinary Medicine, Cairo University, Giza 12211, Egypt; abdelaty44@hotmail.com; 14 Department of Medical Pharmacology, Medical Faculty, Atatürk University, Erzurum 25240, Turkey

**Keywords:** environmental stressors, signal–noise hypothesis, metabolite-mediated regulation, redox homeostasis, nanoplastics, cell signaling

## Abstract

Biological systems maintain functional integrity under environmental stress through tightly coordinated signaling networks, yet the mechanisms that preserve signal fidelity in the presence of stochastic oxidative and ionic fluctuations across taxa remain incompletely understood. Although redox regulation, coupling between reactive oxygen species (ROS) and Ca^2+^, and hormone-mediated feedback loops have been extensively characterized within individual kingdoms, a cross-kingdom framework linking these pathways through metabolite-mediated modulation is lacking. Here, a cross-kingdom compilation of evidence from plant, animal, and microbial systems is presented to examine how specific endogenous and microbe-derived metabolites and plant fructans modulate the fidelity of stress signaling networks. We propose a metabolite-guided signal fidelity hypothesis in which certain specialized metabolites act as molecular stabilizers in the response of organisms to stress. They buffer transient ROS bursts, limit variability in calcium signaling, and strengthen hormone-regulated feedback loops. Together, these functions preserve the signal-to-noise ratio of stress responses, independent of overt toxicity. These insights lay the groundwork for testable predictions and the development of translational strategies in agriculture, biotechnology, and medicine aimed at improving stress tolerance by stabilizing the flow of biological information.

## 1. Introduction

Life depends on the ability to sense, interpret, and respond to environmental change. Across biological kingdoms, this capacity is mediated by conserved molecular signaling architectures that translate external cues into adaptive physiological responses. As previously reported, membrane-associated signaling and redox-regulated pathways shape cellular communication and stress responsiveness, highlighting the importance of spatial organization and signal integration in maintaining physiological homeostasis [1,2,3,4]. In plants, stress signals integrate rapid reactive oxygen species (ROS) bursts, cytosolic Ca^2+^ transients, mitogen-activated protein kinase (MAPK) cascades, and hormonal networks such as abscisic acid (ABA), jasmonic acid (JA), and salicylic acid (SA). These interconnected modules contribute to drought and salinity tolerance, temperature acclimation, immune defense, and systemic adaptation through coordinated local and long-distance signaling [2,3,4,5,6,7,8,9,10]. These pathways interact synergistically or antagonistically at multiple levels, including hormone biosynthesis and transport, receptor signaling, kinase activity, and transcriptional regulation, thereby coordinating growth–defense trade-offs, drought and salinity tolerance, temperature acclimation, immunity, and recovery from stress [2,3,5,6,7]. Accordingly, the ABA-, JA-, and SA-related pathways emphasized here should be considered representative components of an integrated hormonal network rather than isolated regulators of plant stress responses. Additionally, during heat stress, ROS-Ca^2+^ and kinase signaling further interact with the heat shock factor (HSF)–heat shock protein (HSP) network, linking temperature perception to proteostasis, thermotolerance, and systemic acclimation. Heat stress provides a particularly informative example of how conserved stress protection mechanisms interface with kingdom-specific signaling architectures. In plants, elevated temperature alters membrane and protein homeostasis and initiates signaling involving Ca^2+^ influx, ROS production, kinase cascades, hormone regulation, and heat-shock transcription factors (HSFs). These pathways converge on the induction of HSPs and other protective mechanisms that promote proteostasis and acquired thermotolerance [11]. Heat perception can also generate rapid systemic ROS, Ca^2+^, and electrical signals, allowing locally exposed tissues to induce systemic acquired acclimation. At the molecular level, several HSP families are evolutionarily widespread, with HSP70 representing a highly conserved chaperone system across microorganisms, plants, and animals. Nevertheless, conservation of the core proteostasis function should not be interpreted as a mechanism equivalent among kingdoms because heat sensing, HSF repertoires, HSP family organization, and physiological outputs have undergone substantial lineage-specific diversification. Within the signal-fidelity framework proposed here, the heat-shock response therefore provides an example in which adaptive function depends on the coordinated timing and integration of redox, ionic, transcriptional, and proteostasis networks. HSP-mediated maintenance of protein homeostasis may help preserve the functional integrity of signaling components during heat-induced proteotoxic stress, although a direct causal relationship between HSP activity and quantitative signaling fidelity remains to be experimentally established.

Within plants, both endogenous and microbe-derived small molecules can stabilize ROS and Ca^2+^ dynamics, prime MAPK cascades, and fine-tune hormone signaling, thereby increasing the signal-to-noise ratio of stress responses and contributing to systemic adaptation across tissues. For example, ROS generated through respiratory burst oxidase homolog (RBOH) activity can promote Ca^2+^ influx, while Ca^2+^-dependent kinases can be fed back to regulate ROS production, creating a coupled ROS–Ca^2+^ amplification loop that can subsequently engage MAPK and hormone-dependent responses during systemic stress signaling. Beneficial metabolites such as reuterin, short-chain fatty acids (SCFAs), and plant fructans improve cellular communication fidelity, whereas synthetic nanoparticles and plastic-derived compounds act as noise generators, disrupting redox, ionic, and hormonal circuits [6,7,11,12].

Traditionally, stress biology in both plants and animals has treated disorders as biochemical events that include oxidation, membrane rupture or enzyme inhibition. However, many current environmental stressors, including nanoplastics and plastic-derived compounds, rarely cause obvious cytotoxicity. Instead, they subtly disrupt ROS and Ca^2+^ dynamics and interfere with hormonal networks, thereby compromising the accuracy of stress signaling. Quantitative observations revealed that such perturbations can occur at the level of signaling dynamics: under specific stress conditions, the ROS burst amplitude has been reported to increase by approximately 1.5–3.5-fold, whereas the Ca^2+^ transient frequency can change by approximately 20–60% in Arabidopsis (Table 1). These changes indicate that environmental stress can modify not only the magnitude of biochemical responses but also the temporal properties and variability of intracellular signaling.

Consistent with this view, studies on endocrine-active plastics such as bisphenol A (BPA) have demonstrated that synthetic chemicals can disrupt redox balance, hormone receptor signaling, and inflammatory pathways without necessarily inducing overt cytotoxicity, supporting a signal fidelity rather than damage-centric model of stress [13]. In plants, however, exposure to nanoplastics has been shown to induce oxidative bursts, alter antioxidant enzyme activities, and modify the ABA and SA signaling pathways, interfering with early stress perception and systemic acclimation [14,15,16]. Together, these findings support reframing stress as an information fidelity challenge: the ability of plants to discriminate environmental signals from background noise. We propose that environmental stress may introduce variability into biological signaling networks, and the stochastic or non-informative component of this variability can function as noise that reduces the fidelity of information transmission. In this framework, ROS-Ca^2+^ signaling hubs act as conserved integrative nodes, whereas metabolites contribute to stabilizing information flow. The dynamics of how disruptive environmental stressors introduce noise into ROS–Ca^2+^–hormone networks, while metabolites act as stabilizers to restore signal fidelity and systemic acclimation, are illustrated in Figure 1.

Environmental stressors, such as nanoplastics, endocrine-disrupting compounds (EDCs), and metals, can introduce stochastic “noise” into plant stress-sensing systems, disrupting ROS–Ca^2+^ waves, kinase signaling, electrical activity, and hormone signaling networks such as those related to ABA, JA, and SA, among others. Reduced signaling coherence may lead to unsuitable responses or impaired systemic acclimation and can be quantified through changes in temporal variance, cell-to-cell variability, signal amplitude or frequency distributions, and the propagation coherence of ROS–Ca^2+^ dynamics (Table 1). In contrast, plant- and microbiome-derived metabolites can stabilize redox and ion-flux dynamics, restore signal coherence, and support effective stress acclimation. The testable predictions from a signal–noise framework are shown in Figure 1: stress-induced increases in temporal and cell-to-cell variability of ROS and Ca^2+^ signals, a coherence threshold for systemic signal propagation, and a noise-induced adaptation window in which metabolite buffering restores signaling fidelity. These predictions can be experimentally tested using time-resolved live-cell or tissue imaging with genetically encoded Ca^2+^ and ROS/redox reporters combined with genetic or pharmacological perturbations to quantify changes in signal amplitude, frequency, timing, cell-to-cell variability, spatial propagation, and coherence.

**Table 1:** Reactive oxygen species (ROS)- and Ca^2+^-mediated signaling responses to abiotic stress across biological systems.

Biological System	Stress Type	Signaling Component	Quantitative Response	Signal Property Affected	Representative References
*Arabidopsis thaliana*	Cold, salinity	ROS burst amplitude	~1.5–3.5-fold increase over baseline	Amplitude, variance	[17,18]
*Arabidopsis thaliana*	Mechanical/osmotic stress	Ca^2+^ transient frequency	~20–60% increase	Frequency, coherence	[19,20]
Crop plants (various)	Drought	ROS accumulation	~2–4-fold increase	Mean intensity, noise	[5,21,22,23]
Mammalian cells	Heat stress	Cytosolic Ca^2+^ spikes	~1.3–2.5-fold increase	Amplitude, temporal structure	[24,25]
Mammalian neurons	Oxidative stress	Ca^2+^ oscillation variability	CV increase ~25–70%	Variance, fidelity	[26,27]
Yeast	Nutrient stress	ROS signaling	~1.5–3-fold increase	Noise, adaptive threshold	[28,29]
Aquatic organisms	Thermal stress	Ca^2+^ signaling duration	Prolongation by ~30–80%	Temporal integration	[30,31]

The quantitative values shown are illustrative ranges obtained predominantly from controlled laboratory, cell-based, growth chamber, or controlled organismal studies represented by the cited literature; they are not derived from field monitoring or formal meta-analysis. Experimental conditions, including species, tissue or cell type, stress intensity, exposure duration, and analytical methodology, differ substantially among studies. Accordingly, these values should be interpreted as contextual estimates of reported response magnitudes rather than universal physiological reference ranges.

This potential vulnerability extends across kingdoms, as nanoplastics and related synthetic particulates have been reported to interfere with transport, redox, ionic, and endocrine processes in both plants and animals, although the underlying mechanisms and strength of evidence vary substantially among systems. Strikingly, similar failures of stress-signal regulation are observed in human disease, reinforcing the potential cross-kingdom relevance of this framework. Both COVID-19 and Parkinson’s disease (PD) are associated with oxidative stress, mitochondrial dysfunction, and persistent inflammatory signaling [32,33,34]. These conditions converge on a shared pathological axis involving oxidative stress-driven activation of nuclear factor kappa-light-chain-enhancer of activated B cells (NF-κB) signaling, linking redox imbalance to persistent inflammatory transcriptional programs [32,33,34]. NF-κB functions as a redox-sensitive signal amplifier: transient activation supports adaptive immunity and cellular defense, whereas sustained exposure to ROS locks the pathway into a high-gain state that amplifies noise rather than information. Adaptive danger signals are transient molecular events that convey genuine stress information and trigger protective responses. This logic parallels the stress-induced perturbation of ROS-Ca^2+^-dependent signaling networks in plants, but the corresponding molecular components occupy different hierarchical positions within their respective signaling architectures. In animals, NF-κB functions as a downstream transcriptional regulator whose activity integrates inputs from upstream redox-sensitive and kinase-dependent pathways. In contrast, plant MAPK cascades primarily constitute intermediate signal-transduction modules that relay ROS-, Ca^2+^-, receptor-, and hormone-associated signals to downstream transcriptional regulators. Accordingly, a more appropriate functional comparison with NF-κB can be made at the level of plant stress-responsive transcription factor networks, including those of WRKY, NAC, ERF, and related families, which integrate upstream kinase and hormonal signals to control adaptive gene expression programs. For example, Arabidopsis MPK3/MPK6 directly phosphorylates WRKY33, thereby promoting the transcriptional reprogramming of defense metabolism [35]. Thus, the cross-kingdom correspondence proposed here concerns conserved signaling logic, namely, signal integration followed by transcriptional reprogramming, rather than direct molecular homology or one-to-one equivalence among the NF-κB, MAPK, or phytohormone pathways.

Plants and animals share conserved stress signaling architectures centered on ROS-Ca^2+^ feedback, kinase-mediated signal integration, and hormonal regulation, although the molecular components implementing these functions are not necessarily orthologous. These signaling architectures are vulnerable to noise introduction from environmental stressors, which can alter signal dynamics and compromise systemic information transfer (Figure 2).

Small molecules are integral components of signaling networks that govern stress responses in both plants and animals. Beneficial microbial and endogenous metabolites, such as reuterin, short-chain fatty acids, and plant fructans, can increase redox buffering, modulate ion fluxes, and prime defense networks, thereby supporting the reliability of cellular communication [12,36]. These mechanisms provide a rationale for the development of metabolite-based biostimulants, postbiotic interventions, and related biomimetic strategies aimed at reinforcing stress resistance through the modulation of redox, ionic, and hormonal signaling networks. Conversely, synthetic nanoparticles and plastic-derived compounds can perturb these same regulatory systems. For example, foliar exposure to polystyrene nanoplastics in leafy vegetables has been associated with changes in the ABA–ROS–Ca^2+^ signaling pathway involved in stomatal regulation, illustrating how nanoscale contaminants can interfere with interconnected redox, ionic, and hormonal signaling processes. Such perturbations may compromise the reliability of stress-responsive information transfer [37,38,39].

This duality motivates the development of a unifying, systems-level framework, which we propose to explore as the signal–noise hypothesis. Rather than acting solely through overt biochemical toxicity, environmental agents, either beneficial metabolites or synthetic contaminants, reshape resilience by modulating the fidelity of molecular communication. Related investigations have shown that the modulation of oxidative and apoptotic pathways can reshape cellular stress outcomes, emphasizing that stress responses are governed not only by damage magnitude but also by the regulation and timing of redox-dependent signaling cascades [21,40,41]. In this review, the signal–noise hypothesis is defined as the main systems-level framework that describes how environmental or xenobiotic perturbations may alter the reliability of biological information processing. Environmental stress itself is not considered equivalent to noise because a stress stimulus can constitute biologically meaningful information. Rather, noise here refers to stochastic or contextually noninformative variation that decreases the reliability with which information about a biologically relevant input is encoded, propagated, or decoded by downstream signaling networks. Accordingly, signal fidelity refers to the preservation of the biologically relevant input–response relationship during signaling. Importantly, biological variability is not intrinsically noise because temporal dynamics, oscillations, and spatial patterns can encode information. Coherence is therefore used in a restricted operational sense to describe temporal and/or spatial coordination among defined interacting signaling processes that support effective signal propagation and integration, whereas desynchronization denotes disruption of such coordination. At broader organismal or ecosystem scales, we preferentially refer to functional coordination, communication integrity, or system-level integration unless signaling coherence can be explicitly defined. Within this main framework, the metabolite-guided signal fidelity hypothesis represents a nested mechanistic hypothesis suggesting that specific endogenous, plant-derived, or microbial metabolites may constrain information-degrading perturbations through the modulation of redox homeostasis, ROS-Ca^2+^ dynamics, ion fluxes, and regulatory feedback (Figure 3). Thus, the signal–noise hypothesis addresses the general systems-level problem of information degradation under stress, whereas the metabolite-guided signal fidelity hypothesis addresses one proposed class of molecular mechanisms through which signaling reliability may be preserved. In this view, stress biology is referred to as an information processing challenge: metabolites such as reuterin, SCFAs, and fructans increase coherence by modulating redox states, stabilizing ion fluxes, and reinforcing hormonal feedback, thereby increasing the signal-to-noise ratio of cellular networks. Conversely, nanoplastics and plastic-derived compounds introduce stochastic fluctuations into ROS and Ca^2+^, potentially increasing temporal or cell-to-cell variability and degrading signal coherence. Quantitative measures such as the ROS-burst amplitude and variance, Ca^2+^ transient frequency, and propagation coherence provide potential metrics for evaluating these contrasting effects (Table 1). By shifting the informational balance of redox, ionic, and hormonal pathways, these agents influence whether physiological states converge toward stability or progress toward dysfunction, offering a novel framework for explaining conserved vulnerabilities across kingdoms.

Our previous work on endoplasmic reticulum stress in patients with COVID-19 and PD used complementary in vitro and in silico approaches to examine how sustained activation of adaptive stress pathways may perturb protein-folding and stress-response networks [32]. Extending this framework, we integrate cross-kingdom evidence from plants, animals, and microbes, focusing on metabolite signaling, redox dynamics, and nanoscale environmental exposures that contribute to failures in signaling fidelity. Although the proposed signal-fidelity framework is relevant across biological kingdoms, this review is primarily plant-centered. Plant systems provide the principal context for examining environmental stress perception, ROS–Ca^2+^ dynamics, hormonal feedback, systemic signaling, and metabolite-mediated stabilization. Animal and microbial examples are incorporated to evaluate the broader applicability of the proposed signaling principles rather than to establish direct molecular equivalence among kingdoms. To support this integrative and hypothesis-driven framework, we outline the scope and methodology used to curate and compile cross-kingdom evidence.

## 2. Methodology and Scope

This review adopts an integrative, hypothesis-driven synthesis rather than a formal systematic review or meta-analysis. The objective of this study was to identify and connect mechanistic evidence relevant to the conserved principles of environmental signal processing, redox and ROS–Ca^2+^ signaling, hormonal regulation, metabolite-mediated modulation, and adaptive responses across biological kingdoms. Peer-reviewed literature published primarily from 2010 to 2026 was surveyed using Web of Science, Scopus, and PubMed. Searches were structured around combinations of concept groups, including (i) “reactive oxygen species” OR ROS, (ii) Ca^2+^ OR “calcium signaling” OR “calcium signaling”, (iii) redox OR “redox homeostasis”, (iv) hormone* OR phytohormone* OR “hormonal signaling” OR “hormonal signaling”, (v) stress OR “environmental stress” OR abiotic stress OR xenobiotic*, (vi) metabolite* OR “microbial metabolite*” OR fructan* OR “short-chain fatty acid*”, (vii) nanoplastic* OR microplastic* OR nanoparticle* OR “endocrine-disrupting chemical*”, and (viii) signaling OR “signal fidelity” OR “signal noise” OR coherence OR variability OR “cross-kingdom”. The terms were combined as appropriate for the database and biological context rather than using a single fixed search string for all the databases. Citation tracking of key primary studies and relevant reviews was additionally used to identify studies not captured by the initial keyword searches.

Studies were considered eligible when they were peer-reviewed and provided evidence relevant to at least one component of the proposed framework, including experimentally characterized ROS or Ca^2+^ signaling, redox regulation, hormonal or metabolite-mediated signaling, environmental or xenobiotic perturbation, signaling dynamics, adaptive responses, or mechanistically informative cross-kingdom comparisons. Both plant and non-plant systems were included when they contributed evidence concerning conserved signaling logic or provided a mechanistic context for the proposed framework. Studies were not required to investigate “signal fidelity” or “signal noise” explicitly because these terms represent the conceptual framework developed in the present review rather than established standardized experimental categories.

Mechanistic relevance was assessed qualitatively according to whether a study provided evidence connecting a perturbation, metabolite, or stress response to an identifiable molecular or cellular process within the signaling architecture of interest. Greater mechanistic weight was assigned to studies that experimentally linked perturbations to signaling components, pathway activity, temporal or spatial signaling properties, or functional outcomes, whereas purely associative observations were used primarily as contextual evidence and were not interpreted as establishing causality. Studies that were primarily descriptive were therefore not excluded solely on that basis; however, descriptive findings without a clear connection to the signaling mechanisms addressed by the review were not used as primary support for mechanistic conclusions. Non-peer-reviewed material, studies outside the defined conceptual scope, and reports lacking sufficient relevance to the signaling, redox, hormonal, metabolite, or environmental-stress framework were excluded from the core evidence synthesis.

Because the included literature encompasses heterogeneous organisms, stressors, experimental platforms, and outcome measures, no formal quantitative quality scoring or statistical pooling was performed. Findings were instead synthesized qualitatively according to their experimental context and mechanistic relevance. The purpose of this approach was to identify convergent mechanisms and formulate testable hypotheses rather than to provide an exhaustive estimate of effect sizes or a systematic enumeration of all published studies.

## 3. Nanoplastics as Informational Noise Generators in Plant Stress Signaling

Plants rely on precisely orchestrated systemic signaling networks to detect environmental stress and coordinate local and systemic adaptive responses. Central mediators of this systemic coordination are reactive oxygen species (ROS) and cytosolic calcium (Ca^2+^) waves, which propagate across tissues with distinct spatiotemporal dynamics. Experimentally, these dynamics can be monitored in living plant tissues by time-resolved fluorescence imaging using ROS-sensitive probes or reporters and genetically encoded Ca^2+^ indicators, allowing spatial and temporal resolution of signal initiation and propagation [4,8,9,10]. These signals encode information not only in their amplitude but also through frequency modulation, synchrony, phase relationships, and propagation coherence. Consequently, maintaining the fidelity of integrated ROS-Ca^2+^ waves is critical for accurate stress perception and subsequent adaptive decision-making in plants.

The integration of ROS, Ca^2+^, and electrical signals has been demonstrated to facilitate rapid systemic communication following localized stress, emphasizing the necessity of precise temporal coordination and intercellular synchrony [8]. Analogously, glutamate-triggered Ca^2+^ waves, propagated through glutamate receptor-like channels, are known to initiate jasmonate-dependent defense responses, further underscoring the functional importance of signal coherence in effective systemic signaling [9]. In Arabidopsis, wound-released glutamate activates glutamate receptor-like channel-dependent long-distance Ca^2+^ signaling and downstream defense responses [9]. Although ROS-Ca^2+^-electrical systemic signaling is broadly conserved across plants [3,4,8,10], the precise mechanism involving glutamate–glutamate receptor-like channels has not been demonstrated with equivalent molecular resolution across all species.

Within this conceptual framework, nanoplastics may be understood not only as agents of oxidative stress but also as generators of informational noise that impairs the coherence of ROS-Ca^2+^ wave dynamics. By introducing stochastic fluctuations, temporal delays, and cellular desynchronization into stress-triggered signaling cascades, nanoplastics can theoretically impair signal fidelity even in the absence of overt cytotoxicity. The following sections explore how such noise-driven perturbations disrupt plant stress signaling networks and compromise systemic adaptation capacity.

This framework yields several testable predictions in plant systems. First, exposure to nanoplastics is predicted to increase cell-to-cell and temporal variance in Ca^2+^ and ROS signals without proportionally shifting mean signal levels. Second, systemic stress signals, including ROS, Ca^2+^, and electrical waves, are expected to exhibit reduced propagation coherence and delayed or attenuated distal transcriptional responses. Third, this hypothesis could be tested in crops by combining the supplementation of metabolites, such as fructan and melatonin, with time-resolved measurements of ROS, Ca^2+^, and hormonal signaling during controlled stress exposure, thereby distinguishing general improvements in stress tolerance from direct preservation of signaling fidelity.

These predictions motivate the following section, which examines nanoplastics and other synthetic stressors as disruptive modulators that introduce informational noise into plant signaling networks.

Nanoplastics (<1 µm in size) are emerging abiotic stressors that disrupt biological information flow rather than acting primarily through overt toxicity or osmotic imbalance. Following adhesion to the root surface, nanoplastics may penetrate through cell-wall pores or discontinuities and move through the apoplast, while cellular internalization has also been proposed to involve plasmodesmata, endocytosis, membrane transporters, and aquaporins; foliar uptake may additionally occur through stomatal openings [42,43,44]. Plants perceive nanoplastics indirectly through disturbances in redox balance, ion flux, and hormonal homeostasis. Exposure to nanoplastics triggers the overproduction of ROS, cytosolic Ca^2+^ oscillations, and membrane depolarization, which are mediated by NADPH oxidases, peroxidases, mechanosensitive channels (MSLs, MCAs), and cyclic nucleotide-gated channels (CNGCs) [42,45]. Hormonal signaling, particularly by ABA, JA, and SA, is dysregulated, resulting in the activation of drought responses and defense pathways [43]. Collectively, these effects introduce stochastic fluctuations that degrade the fidelity of stress perception and systemic signaling, exemplifying how synthetic stressors function as noise generators within conserved molecular networks. Representative examples of nanoplastic-induced perturbations across major plant stress signaling modalities are summarized in Table 2. These examples are based on species-specific experimental studies, including those on *Arabidopsis thaliana*, maize, and other plant systems, and should therefore be interpreted within the biological context of the cited studies rather than as universally conserved responses.

**Table 2:** Species-specific examples of nanoplastic-associated perturbations of plant signaling under (a)biotic stress.

Signal Type	Typical Trigger	Nanoplastic Effect	Potential Receptors/Sensors	References
ROS burst	Pathogen, salinity	Elevated oxidative load	NADPH oxidases, peroxidases	[42]
Ca^2+^ wave	Mechanical, cold	Altered cytosolic Ca^2+^	MSL, MCA channels	[45]
Hormonal flux	Drought, wounding	ABA/ethylene imbalance	PYR/PYL, ETR receptors	[46]
Electrical signaling	Heat, touch	Membrane depolarization	GLR, CNGCs	[37,38]

CNGC, cyclic nucleotide-gated channel; ETR, ethylene response receptor; GLR, glutamate receptor-like channel; MSL, mechanosensitive channel of small conductance-like channel; MCA, mid1-complementing activity channel; PYR/PYL, pyrabactin resistance/pyrabactin-resistance-like receptor group; ROS, reactive oxygen species.

Beyond initial uptake, nanoplastics can enter vascular tissues and undergo systemic translocation through xylem- and phloem-associated transport. For example, 50 nm polystyrene nanoparticles have been detected intracellularly in plant roots, while nanoplastic particles have also been detected in the apoplast and xylem [42]. Root-to-shoot translocation is influenced by transpiration-driven water flow as well as by particle size, aggregation state, surface properties, exposure duration, growth medium, and plant species [42,43]. Although these observations establish vascular transport, quantitative rates of nanoplastic movement through the xylem remain insufficiently resolved. Such a systemic distribution may increase the spatial extent over which nanoplastics perturb redox, ionic, and hormonal homeostasis, potentially compromising long-distance stress communication. Under controlled conditions, experiments have shown that the constant activation of defense metabolism under non-stress conditions wastes resources, whereas the suppression of ABA responses compromises drought resilience [46]. Nanoplastics also interfere with the transport machinery; they can adsorb onto or block aquaporins [47]; deactivate ion channels such as CNGCs, glutamate receptor-like channels (GLRs), and two-pore channel 1 (TPC1) [45]; and hinder vesicular trafficking or ABC transporters, thereby affecting hormone distribution and defense compounds. The dynamics of these mechanisms, whether cumulative or reversible, are contingent upon the specific stressor agent and exposure time. Comparable systems-level vulnerability is observed in animals, although the underlying anatomical structures are distinct. Nanoplastics can acquire protein coronas that modify their biological identity and interactions with surrounding biomolecules [48,49], while systemic exposure is associated with disruption of vascular and cellular homeostasis, oxidative imbalance, Ca^2+^ dysregulation, and inflammatory responses [37,38,50]. In plants, nanoplastic entry into xylem- and phloem-associated transport pathways can likewise perturb vascular transport and long-distance signaling [42,43,50]. Thus, plant vascular coherence and animal endothelial integrity represent functionally analogous points of vulnerability: disruption of either interface can propagate local physicochemical stress into organism-wide disturbances in transport, redox regulation, ionic homeostasis, and intercellular communication. Conceptually, nanoplastics act as synthetic channel modulators, corrupting the same molecular circuits that govern stress sensing and systemic response. Their ability to reduce informational fidelity places them within a new category of abiotic stressors.

To complement such qualitative observations, quantitative analyses across diverse biological systems revealed conserved patterns of ROS- and Ca^2+^-mediated signaling disruption under environmental stress. Stressors consistently increase the variance, amplitude, or temporal distortion of redox and ionic signals without necessarily altering the mean levels (Table 1). The conserved modulation of signal properties, whether in Arabidopsis, crop plants, mammalian cells, neurons, yeast or aquatic organisms, underscores the cross-kingdom vulnerability of ROS-Ca^2+^ hubs to noise injection. Collectively, these experimentally reported changes in the magnitude and temporal properties of ROS and Ca^2+^ signaling provide an empirical context for the signal–noise hypothesis, although direct quantitative comparisons across biological systems remain constrained by differences in experimental design and measurement methodology.

In crop systems, identifying metabolites and regulatory nodes that stabilize ROS, Ca^2+^, and hormonal dynamics may guide the development of biostimulants, breeding, and metabolic engineering strategies aimed at improving resilience to environmental stress [51,52,53]. In support of this view, metabolomic analyses of bioactive oligosaccharides (deduced to be composed of six monosaccharides with a molecular weight of approximately 880 Da) have demonstrated that small metabolites can reorganize metabolic and microbial networks, improving redox balance and inflammatory homeostasis at the organismal level, illustrating how metabolites stabilize physiological networks beyond direct stress mitigation [54].

## 4. Plant and Microbial Metabolites as Cross-Kingdom Stabilizers of Stress Signaling

In contrast to the disruptive effects summarized in Table 1, microbial and plant metabolites function as conserved stabilizers of stress signaling, buffering redox fluctuations and restoring coherence in ROS-Ca^2+^-hormone networks across kingdoms. The vulnerability associated with exposure to nanoplastics highlights the importance of cross-kingdom mechanisms that stabilize stress signaling.

Microbial metabolites such as reuterin (3-hydroxypropionaldehyde), produced by *Limosilactobacillus reuteri*, can modulate redox and stress-response pathways, but their effects are strongly dependent on concentration, exposure duration, and biological context. Reuterin can perturb glutathione metabolism and redox balance in *Escherichia coli* and generate ROS in *Clostridioides difficile*, effects that can be counteracted by reduced glutathione [55,56]. Reuterin also inhibits catalase activity in *Aspergillus flavus*, leading to ROS accumulation [57], whereas in host macrophages, it can attenuate lipopolysaccharide (LPS)-induced oxidative stress through activation of the Nrf2/HO-1 pathway [58]. Thus, reuterin illustrates the dual nature of microbial metabolites: at appropriate concentrations and in specific host contexts, they may contribute to redox and inflammatory regulation, whereas higher or context-dependent exposure can itself become a source of oxidative or metabolic stress.

SCFAs, such as butyrate and propionate, similarly increase antioxidant capacity and modulate inflammatory gene expression, linking microbial metabolic output to host signal stability. SCFAs, end products of dietary fiber fermentation, act through multiple mechanisms: (i) inhibition of histone deacetylases, regulating epigenetic control of inflammatory genes [59]; (ii) modulation of inflammasome activity and G protein-coupled receptor signaling in macrophages [60]; and (iii) functioning as unique acyl-lysine histone marks, directly coupling microbial metabolism to transcriptional programs [61]. Together with reuterin, SCFAs demonstrate how microbial metabolites converge on redox and transcriptional pathways to stabilize signaling dynamics. Comparable buffering strategies are also evident in animal systems, where engineered biopolymer conjugates can be used to modulate stress-related signaling and tissue responses. For example, an engineered chitosan–gentamicin conjugate (CS-GT) attenuated heat stress-induced intestinal barrier injury by restoring tight junction integrity and inhibiting aberrant activation of the TLR4/STAT6/MYLK pathway [62]. Mechanistically, CS-GT reversed decreases in transmembrane resistance, reduced permeability, and upregulated occludin and claudin proteins, thereby contributing to the restoration of barrier function during stress [62]. This animal model illustrates how engineered biopolymers can function as “stabilizers of information”, buffering against stochastic noise in stress pathways much like microbial or plant metabolites do in plants.

Such cross-kingdom parallels reinforce the signal–noise hypothesis, in which the preservation of signaling fidelity may contribute to physiological resilience by reducing maladaptive responses to environmental stress. Melatonin provides an additional and particularly informative cross-kingdom example of an endogenous small molecule that couples redox regulation with stress signaling. In animals, melatonin is a well-established endocrine indoleamine involved in circadian organization, metabolic and reproductive regulation, stress adaptation, and antioxidant defense [63]. Microbial melatonin-producing symbionts have been implicated in the improvement of plant responses to (a)biotic stress [64]. In plants, endogenous melatonin functions as a pleiotropic signaling molecule with phytohormone-like properties and contributes to tolerance to drought, salinity, temperature extremes, heavy metals, and pathogen challenge. Its protective activity extends beyond direct radical scavenging and includes the regulation of antioxidant enzymes, ROS homeostasis, ion balance, stress-responsive gene expression, and extensive crosstalk with phytohormones such as ABA, auxin, cytokinins, ethylene, JA, and SA [65]. Importantly, melatonin also directly interacts with the ROS–Ca^2+^ signaling architecture emphasized in this review. In Arabidopsis, melatonin-induced stomatal closure has been associated with CAND2/PMTR1-dependent H_2_O_2_ production and Ca^2+^ influx [66], whereas more recent evidence links PMTR1, GPA1, and the H_2_O_2_ sensor HPCA1 to drought-responsive stomatal signaling [67]. These observations position melatonin as a potential stabilizer and integrator of redox, ionic, and hormonal information flow. Nevertheless, this cross-kingdom correspondence should be interpreted at the level of functional signaling logic rather than direct molecular equivalence because melatonin perception is well established through MT1/MT2 receptor systems in animals, whereas the biochemical identity and signaling properties of plant melatonin receptors remain incompletely resolved.

Plant-derived fructans, including inulin and related β-(2,1)-linked polymers, and reducing carbohydrates contribute to stress tolerance by stabilizing redox and hormonal homeostasis. Under abiotic stress, fructans and other carbohydrates (fructose, glucose, sucrose and myo-inositol) increase the activities of antioxidant enzymes such as superoxide dismutase (SOD), catalase (CAT), and ascorbate peroxidase (APX), limiting ROS accumulation [68,69,70], and interact with hormonal networks to balance ABA levels [71]. By buffering stochastic ROS fluctuations, fructans increase the reliability of transcriptional and developmental responses, acting as molecular stabilizers rather than direct growth regulators.

A functional parallel can be drawn between plant cytokinins and animal cytokines insofar as both participate in the coordination of stress-responsive physiological networks, although their molecular mechanisms are distinct. In *Oryza sativa* seedlings, exogenous kinetin directly modulates ROS homeostasis and antioxidant enzyme activity under heat stress [51]. Similarly, root-derived trans-zeatin-type cytokinins promote cold tolerance by regulating energy metabolism and cellular energy homeostasis, an effect that can also be enhanced by exogenous trans-zeatin supplementation [52]. These findings support a direct role for cytokinin signaling in redox and metabolic adjustment during stress. Root endophytes can additionally modulate plant H_2_O_2_ homeostasis through a separate plant–microbe interaction mechanism [72]. However, the available evidence does not establish cytokinin signaling as a mediator of this endophyte-dependent response. Thus, cytokinin signaling and endophyte-mediated redox regulation should be regarded as complementary mechanisms contributing to plant stress resistance. However, cytokinin activity should not be interpreted as uniformly protective [73]. Under water deficit, active cytokinin pools and long-distance cytokinin transport are frequently reduced, and the physiological consequences of altering cytokinin metabolism depend on stress intensity and duration, tissue and developmental context, and crosstalk with ABA and other hormones. Controlled cytokinin reduction can favor water conservation and modify root–shoot allocation, whereas spatially or temporally restricted maintenance of cytokinin activity may preserve photosynthetic competence, delay senescence, and facilitate poststress recovery. Thus, cytokinin-mediated stress adaptation reflects the dynamic reconfiguration of an interacting hormonal network rather than the independent action of a single protective hormone. Brassinosteroids provide a complementary example of this network behavior. They contribute to adaptation to drought, salinity, temperature extremes, and other environmental stresses by modulating antioxidant defenses, metabolism, growth–stress trade-offs, and stress-responsive transcription [7]. These effects arise partly through extensive interactions with ABA, JA, SA, cytokinins, auxin, ethylene, and gibberellins. Mechanistically, convergence between brassinosteroids and ABA pathways at components such as BRASSINOSTEROID-INSENSITIVE 2 (BIN2) and SnRK2-related signaling illustrates how hormonal pathways can share regulatory nodes rather than operate as parallel independent channels [74]. Consequently, within the signal-fidelity framework proposed here, brassinosteroids and cytokinins are best viewed as components of a dynamic phytohormonal interactome coupled to ROS-Ca^2+^-kinase signaling. The direction and magnitude of their contribution to stress adaptation depend on the identity, intensity, duration, and developmental context of the stressor.

Acknowledging metabolites as “stabilizers of information” opens translational opportunities. Engineering plant–microbe consortia to increase fructan production, such as overexpressing wheat fructan biosynthesis genes, may help plants buffer pollutant-induced noise and environmental stress [53]. In animals, probiotic or postbiotic interventions using *Lactobacillus reuteri*, as well as postbiotic approaches based on microbial metabolites, can reinforce microbial redox buffering. Indeed, supplementation with reuterin in piglets reshaped the plasma metabolome and was associated with improved resilience, suggesting systemic redox homeostasis [75]. Together, these strategies feature metabolic symbiosis, in which microbes and their metabolites work together as a highly conserved mechanism for maintaining stress signaling fidelity in increasingly noisy environments (Figure 4).

## 5. Signal–Noise Hypothesis: A Main Systems-Level Framework for Stress-Associated Perturbation of Biological Information Processing

We propose that environmental and xenobiotic stressors can affect biological systems not only through direct biochemical damage but also by perturbing the processing of biological information. Within the present framework, such perturbations constitute noise when they introduce stochastic or contextually noninformative variation, distort informative signaling dynamics, or disrupt temporal and spatial coordination sufficiently to reduce reliable stimulus–response coupling. This distinction is important because increases in ROS, Ca^2+^ fluctuations, or signaling variability should not themselves be interpreted as loss of fidelity; structured temporal and spatial dynamics may constitute information-bearing components of the response. The signal–noise hypothesis therefore predicts loss of fidelity, specifically when stress-associated perturbations reduce reliable encoding, propagation, integration, or decoding of biologically relevant signals [76].

Fluctuations in signaling dynamics can have profound consequences across biological systems. In animal systems, these fluctuations have been associated with mitochondrial dysfunction, disrupted signaling cascades, and endocrine imbalance [77].

In plants, similar disturbances manifest as desynchronized ROS-Ca^2+^ oscillations and deregulated hormonal crosstalk involving ABA, JA, and SA, ultimately impairing systemic root-to-shoot communication [42,43]. Building on these mechanistic observations, a systems biology perspective frames biochemical networks as constrained information transmission systems. Stressors that increase stochastic fluctuations reduce mutual information between the stimulus and response, lowering the effective signal-to-noise ratio and compromising adaptive responses [78].

Stochastic fluctuations inherent to biochemical reactions can degrade the information transmission capacity of signaling pathways, reducing effective signal-to-noise ratios and limiting reliable stimulus–response coupling in cell networks [76]. Such informational degradation manifests differently across kingdoms. In plants, systemic Ca^2+^ and ROS waves are established components of rapid whole-organism stress signaling, particularly in model systems such as *Arabidopsis thaliana* [8,9]. Nanoplastic-induced perturbations of redox and stress signaling have been reported in several plant species, including maize (*Zea mays*) [79], although direct disruption of the spatiotemporal coherence of systemic ROS–Ca^2+^ waves by nanoplastics remains to be experimentally demonstrated. In animals, environmental noise and pollution alter endocrine outputs such as testosterone and corticosterone, which is indicative of disturbed hormonal regulation under chronic sensory stress [80]. Collectively, these observations support the signal–noise hypothesis, in which toxicological outcomes arise not only from direct molecular damage but also from disrupted coordination and reliability within signaling networks.

The signal–noise hypothesis yields testable predictions, such as the following: (i) stressors increase the variance in signaling modalities (ROS, Ca^2+^ fluxes, and hormonal oscillations) without proportionally altering the mean levels, thereby amplifying noise; (ii) mutual information between stimuli and cellular responses declines, reflecting impaired information transfer and reduced decoding accuracy; (iii) signal desynchronization is correlated with functional impairments, such as delayed systemic responses in plants or mitochondrial and endocrine dysfunction in animals; and (iv) interventions that buffer the redox state, stabilize ion fluxes, or normalize hormonal feedback loops should restore signaling coherence, even under persistent stress exposure.

Together, these predictions highlight how noise manifests across scales, from molecular signaling to organismal behavior. By integrating mechanistic studies with quantitative signaling theory, this perspective introduces a novel conceptual lens through which signal noise emerges as a cross-kingdom unifying principle of stress biology. This framework explains how diverse stressors compromise biological resilience not only through direct structural damage but also by corrupting the informational integrity of signaling networks, thereby undermining the ability of organisms to distinguish genuine cues from background variability (Figure 5).

## 6. Sensory and Systemic Vulnerabilities: From Molecular Noise to Organism-Level Disruption

Extending beyond molecular signaling, the consequences of stochastic interference become evident at the sensory and systemic levels. As mentioned above, plants and animals rely on conserved architectures such as membrane-tension-sensitive ion channels, regulated Ca^2+^ fluxes, and hormonally coordinated networks to transduce mechanical and chemical stimuli into adaptive responses. In plants, mechanosensitive channels such as MSL and MCA proteins mediate responses to touch, osmotic stress, and membrane tension [81,82], integrating with ROS signaling and hormonal regulation to form a nexus of mechanical and redox-based perception [83]. Mechanically induced Ca^2+^ influx can feed into Ca^2+^-dependent regulation of RBOH/NADPH oxidases, promoting ROS production that, in turn, further modulates Ca^2+^ signaling and reinforces the coupling between mechanoperception and redox responses [81,83]. Exposure to nanoplastics may impair these pathways through membrane embedding or by altering biophysical properties, leading to erratic Ca^2+^ spikes, stochastic ROS accumulation, and hormonal misfiring.

In parallel, animals show disruption of mechanotransduction: Piezo-type mechanosensitive ion channel component 1 (PEZO-1) channels are critical for sensing mechanical stimuli, and recent work in *Caenorhabditis elegans* has demonstrated that nanoplastics impair neuronal and behavioral functions via PEZO-1, implicating mechanosensitive gating and oxidative signaling in organismal responses [84].

Chemical perception is similarly vulnerable. Plants rely on volatile organic compounds (VOCs), such as methyl jasmonate, green leaf volatiles and ethylene, to orchestrate defense, development, and pollinator attraction [85]. Nanoplastics and plastic-derived volatiles may interfere with these systems: microplastic exposure alters biogenic emission profiles in lettuce, increasing stress-related aldehydes and ketones [86]. By mimicking or masking native volatiles, synthetic VOCs perturb ligand–receptor interactions, desynchronize hormonal axes, and induce false alarms in chemical signaling, which is consistent with evidence that nanomaterials disrupt phytohormonal crosstalk [87].

As illustrated in Figure 6, molecular disruptions increase in scale to ecosystem consequences, where altered VOC emissions impair pollinator attraction, rhizosphere communication is destabilized, and endocrine imbalance in animals leads to reproductive and behavioral disorders.

In animals, pheromonal and neurotransmitter signaling are likewise susceptible to contamination and chemical interference, with synthetic VOCs potentially altering ligand recognition and neuroendocrine coordination. These effects are mechanistically plausible because of the sensitivity of chemical-receptor systems to exogenous ligands, but their direct contribution to signal desynchronization in vertebrates requires further experimental validation. Metabolic and reproductive signaling axes are also affected. In plants, the sucrose nonfermenting-1-related protein kinase 1-target of rapamycin (SnRK1–TOR) antagonistic system coordinates root–shoot communication, nutrient allocation, and flowering timing [87,88]. The SnRK1–TOR module is also functionally coupled to redox status, as stress-induced changes in ROS and cellular energy availability converge on growth–autophagy decisions governed by these antagonistic kinases [87,89]. Nanoplastics disrupt nutrient transporters, adsorb ions, and misplace hormone carriers, resulting in false starvation signals, desynchronized reproductive timing, and aberrant developmental outcomes. For example, nanoplastics derived from polyethylene terephthalate (PET) alter potassium accumulation in foxtail millet (*Setaria italica*) via ROS signaling, interfering with ion homeostasis [90]. Direct evidence that nanoplastics modify SnRK1 or TOR activity in plants is currently lacking. Therefore, the involvement of the SnRK1–TOR axis should presently be considered a hypothesis arising from known nanoplastic-induced perturbations in ROS, ion, nutrient, and metabolic homeostasis [89]. Reproductive processes such as pollen tube guidance, which depend on finely tuned chemical–electrical gradients, may also be vulnerable, although empirical validation is needed. These signaling perturbations create vulnerabilities that extend into deeper physiological systems, particularly reproductive and developmental networks.

In animals, chronic polystyrene microplastic exposure reduces testosterone levels by disrupting the luteinizing hormone (LH)-luteinizing hormone receptor (LHR)/cyclic AMP/protein kinase A (PKA)/steroidogenic acute regulatory protein (StAR) pathway and induces oxidative stress and apoptosis in Leydig cells [91,92]. These exposures also activate broader signaling networks, including the mTOR, MAPK, and phosphatidylinositol 3-kinase (PI3K)/protein kinase B (AKT) pathways, which contribute to reproductive dysfunction. Plastic-derived endocrine disruptors such as BPA have been reported to perturb AMPK/mTOR/ULK1 signaling in ovarian granulosa cells, with evidence from both human granulosa cells in vitro and mouse models in vivo, potentially contributing to impaired ovarian function and oocyte maturation [93]. Nanoplastics also affect female fertility by altering the ovarian structure, hormone levels (LH and FSH), and oocyte viability, ultimately reducing reproductive success. Exposure to maternal microplastics can trigger placental stress via the GRP78/IRE1α/JNK axis (a central component of the unfolded protein response (UPR)), leading to placental dysfunction and embryonic growth retardation [94].

In addition to nanoplastics and plastic-derived compounds, other environmental stressors, such as heavy metals (Cd and Pb) and endocrine-disrupting compounds (EDCs), can perturb signaling networks through distinct mechanisms. In plants, heavy metals alter ROS and Ca^2+^ signaling, MAPK cascades, and transporter localization, whereas in animals, EDCs can mimic or antagonize endogenous hormones and alter endocrine feedback regulation [45,77,94,95].

In animals, EDCs mimic or antagonize natural hormones, desynchronizing endocrine feedback loops and amplifying variability in reproductive signaling [77]. For example, experimental coexposure of human KGN granulosa cells to polystyrene nanoplastics and triclosan resulted in synergistic cytotoxicity associated with increased ROS accumulation, illustrating how combined environmental stressors can amplify oxidative perturbation [96].

These perturbations across kingdoms demonstrate that the systemic consequences of environmental signal disruption extend beyond transient biochemical fluctuations to developmental and reproductive outcomes. In humans, increasing evidence of microplastic and nanoplastic exposure has raised concerns regarding potential reproductive and developmental effects, although direct epidemiological evidence linking specific exposure levels to impaired signaling fidelity remains limited. Experimental and human-relevant studies have reported associations with reproductive toxicity, infertility-related outcomes, and developmental abnormalities, supporting the need for epidemiological studies that integrate exposure assessment with molecular and physiological endpoints [97,98]. In plants, micro- and nanoplastics can impair germination, growth, and development through oxidative stress and disruption of nutrient and ion transport [99]. Future research should directly examine SnRK1–TOR dynamics, hormone transporter localization, and pollen tube guidance under nanoplastic stress in plants as well as under multiple stressor exposures in animals to resolve key mechanistic gaps and strengthen the understanding of how environmental signal noise reshapes organismal function.

## 7. Cross-Kingdom Mechanistic Homology and Evolutionary Implications

By building on the sensory and systemic vulnerabilities outlined above, cross-kingdom comparisons reveal that these disruptions reflect highly conserved signaling architectures and evolutionary constraints. While many environmental stressors impact conserved signaling across kingdoms, plants offer particularly informative models for understanding how environmental noise perturbs sensing, integration, and systemic responses.

Plants rely on highly integrated sensing networks to coordinate growth, development, and stress responses. Central to this integration are Ca^2+^-ROS oscillations, kinase cascades, and hormone feedback loops such as those involving auxin, ABA, and the SnRK1-TOR nutrient-sensing axis [89]. For example, localized wounding triggers an ROS burst that propagates as an electrical-hydraulic wave, synchronizing defense and repair responses across distant tissues [9,10]. Similar ROS-Ca^2+^ signaling architectures operate under abiotic stresses such as salinity, drought, and heavy metal exposure, preserving systemic coherence and buffering against stochastic fluctuations introduced by environmental stressors, including nanoplastics, heavy metals, and chemical volatiles [4,8,100,101]. These systemic modules exemplify how plants maintain signal fidelity, effectively filtering environmental “noise” and ensuring coordinated responses to diverse cues, including light, touch, temperature, and nutrient availability.

To maintain systemic coherence, animals employ functionally analogous motifs to integrate metabolic, stress, and reproductive cues, reflecting a deeply conserved architecture across kingdoms. Animals share homologous signaling motifs, including Ca^2+^ influx, ROS waves, kinase cascades, and hormone-regulated feedback loops, demonstrating the evolutionary conservation of core regulatory architectures [76]. In these networks, ROS and Ca^2+^ dynamics function as central integrators of metabolic and stress information, shaping downstream kinase activity and transcriptional responses. In particular, the AMPK-mTOR axis operates analogously to the plant SnRK1-TOR system: Ca^2+^ transients and ROS fluctuations modulate AMPK activity, which in turn tunes mTOR-dependent energy sensing, endocrine feedback, and reproductive readiness. Stochastic perturbations can be quantified by measuring changes in signal variance, coefficient of variation, temporal autocorrelation, cell-to-cell variability, and spatial propagation coherence of ROS and Ca^2+^ dynamics, together with changes in the relationship between stimulus intensity and downstream response.

The functional analogy between plant SnRK1-TOR and animal AMPK-mTOR suggests that perturbations in redox, ionic, and metabolic homeostasis could converge on these energy-sensing systems [77,96]. However, the strength of evidence for direct nanoplastic-dependent regulation differs between kingdoms, and architectural conservation should not be interpreted as evidence of an identical toxicological mechanism.

Modern environmental stressors exploit these conserved signaling architectures in highly specific ways. For example, nanoplastics can increase ROS levels and disturb Ca^2+^ dynamics while also affecting membrane microdomains, lipid–protein interactions, vesicular trafficking, and transporter polarity. These effects may alter the localization of nutrient and hormone transporters and introduce stochastic fluctuations into conserved regulatory hubs. Whether such membrane and trafficking perturbations are reversible after removal of the stressor or become persistent with repeated or prolonged exposure remains insufficiently resolved and should be addressed through longitudinal studies. In plants, these perturbations may destabilize SnRK1–TOR decision-making, distort source–sink allocation, impair systemic wound-response propagation, and compromise long-distance hormonal transport. In animals, analogous effects may influence AMPK–mTOR activity, steroidogenic enzyme compartmentalization, neuroendocrine feedback timing, and receptor trafficking.

From an evolutionary perspective, these shared vulnerabilities arise because ROS/Ca^2+^ signaling, kinase-cascade logic, and hormone-mediated feedback loops were established and conserved early in multicellular evolution. The early conservation of ROS/Ca^2+^-based signaling provided an efficient and reusable architecture for environmental sensing and rapid coordination, but conservation of these core modules may also constrain subsequent diversification of signal-processing logic. Consequently, perturbations that alter ROS production, Ca^2+^ dynamics, or their coupling can affect signaling architectures that remain functionally important across diverse lineages. The systemic effects of such stressors, including metabolic imbalance, impaired reproduction, and disrupted development, may therefore reflect not only chemical or physical toxicity but also vulnerability arising from a conserved signaling architecture [4,101]. Understanding these cross-kingdom homologies through a plant-centric lens offers a mechanistic framework for predicting how synthetic and natural stressors reshape organismal resilience across the tree of life while emphasizing plants as a sentinel system for studying signal fidelity under environmental noise.

## 8. Systemic and Ecological Consequences

The systemic effects of informational noise generated by nanoscale pollutants extend far beyond cellular dysfunction and manifest across ecological networks that depend on precise biochemical and mechanical communication. Many cross-kingdom interactions, including plant–pollinator signaling, microbial symbioses, predator–prey dynamics, and social behavior in animals, affect the high-fidelity transfer of chemical cues, VOCs, pheromones, quorum-sensing molecules, and mechanosensory inputs. Nanoplastics and other ultrafine particulates act as exogenous informational nodes, perturbing these communication systems through adsorptive interference, oxidative modulation, and noise amplification within shared signaling channels.

In terrestrial ecosystems, the adsorption of floral VOCs onto nanoplastic or plastic-derived surfaces has been proposed as a mechanism that could reduce volatile dispersion or alter signal composition, potentially affecting pollinator attraction and plant reproductive success [102]. Nanoplastics also disrupt the metabolic dialog between plants and their rhizosphere microbiota. Changes in microbial metabolite profiles, including reductions in the expression of auxins, siderophores, and quorum-sensing autoinducers, impair nutrient sensing, root development, and immune priming [44,103]. These perturbations weaken the stabilizing feedback loops that normally balance plant growth and defense, shifting ecosystems toward reduced productivity and resilience.

Aquatic and terrestrial animals likewise exhibit behavioral and physiological desynchronization in response to nanoplastic-induced signaling disturbances. Endocrine-active plastics modulate hormone receptor dynamics, leading to alterations in circadian timing, reproductive signaling, and stress-axis regulation. Structural integration of nanoparticles into mechanosensory tissues disrupts lateral line communication in fish and alters vibrational cues in insects, decreasing foraging efficiency, predator avoidance, and mate recognition [103]. Experimental studies have reported the sensory and behavioral effects of micro- and nanoplastic exposure in aquatic animals; however, the extent to which these effects occur at environmentally relevant concentrations remains an important unresolved question [84]. In social species, the distortion of semiochemical cues further decreases group cohesion and cooperative behaviors, which are essential for population stability.

Viewing ecosystem dysfunction through the lens of signal fidelity rather than pollutant concentration provides a more unified mechanistic framework for the early detection of ecological stress. Because informational networks degrade before overt population declines, metrics derived from redox oscillation amplitude, intracellular Ca^2+^ wave consistency, and mutual information mapping between interacting species offer sensitive, cross-kingdom indicators of ecological disturbance [78,104]. By quantifying how pollutants alter the structure, timing, and reliability of biological communication, these approaches enable the predictive modeling of ecosystem tipping points and support interventions aimed at restoring communication integrity.

## 9. Translational and Biotechnological Applications

A mechanistic understanding of how nanoplastics generate informational disruption, from altered redox signaling to degraded chemical and mechanical communication, enables the development of targeted biotechnological strategies aimed at restoring signal fidelity across biological systems. In plants, approaches that reinforce mechanosensitive channel stability (e.g., MSL- and MCA-family channels), enhance ROS-buffering networks or modulate vibrational priming can improve the robustness of cellular decoding mechanisms under environmental noise [14,42,105].

The exogenous application of stabilizing volatiles, antioxidants, or phytoactive small molecules has been shown to attenuate oscillations in signaling pathways, preserving stomatal dynamics, photoreceptor tuning, and root developmental patterning under pollutant stress. Antioxidant-based interventions represent a potential strategy for buffering against pollutant-induced oxidative and signaling disturbances, although their effectiveness under realistic environmental conditions and at the field scale requires further validation. Rhizosphere-derived bacterial consortia capable of degrading or transforming micro- and nanoplastics have recently been demonstrated under controlled conditions, supporting the feasibility of microbe-based mitigation strategies in soil environments. For example, rhizospheric consortia enriched from plastic-polluted soils have shown significant PET depolymerization efficiency [106], whereas microbial assemblies can biodegrade and transform plastic residues into soil [107]. In parallel, plant growth–promoting rhizobacteria have been shown to mitigate microplastic-induced stress by reshaping root-associated microbiomes, enhancing antioxidant responses, and stabilizing key physiological processes in plants [108].

In animals, probiotic, symbiotic and postbiotic formulations represent potential translational strategies for restoring physiological coordination following environmental stress. Controlled animal studies have shown that microbial or postbiotic supplementation can alter host metabolic and microbial profiles; for example, *Lactobacillus reuteri* postbiotic supplementation in weaned piglets reshaped the plasma metabolome and was associated with improved physiological performance [75]. However, direct evidence that these interventions restore signaling fidelity specifically following nanoplastic exposure remains limited, and their efficacy against nanoplastic-induced oxidative, endocrine, or neuroimmune disturbances requires targeted experimental validation [109].

Together, these interventions on plants, microbes, and animals illustrate a synthetic ecological framework in which restoring communication fidelity rather than simply removing toxicants is the central principle of environmental resilience engineering. By targeting the signaling bottlenecks most vulnerable to informational noise, such approaches enable cross-scale stabilization of biological networks and provide actionable translational strategies for mitigating the ecological and physiological consequences of nanoscale pollutants [78]. These cross-kingdom strategies (Figure 7) integrate plant-, microbiome- and animal-centered interventions under a unifying framework of signaling fidelity restoration.

## 10. Outstanding Questions and Future Directions

Despite growing evidence that environmental perturbations alter ROS, Ca^2+^, kinase, hormonal, and metabolic signaling across biological kingdoms, several questions must be answered before signal fidelity can be established as a quantitatively comparable property of stress–response networks. The first challenge concerns how signal fidelity should be measured across heterogeneous biological systems. A single universal numerical indicator is unlikely to capture the diversity of signaling codes because biological information can be encoded through amplitude, frequency, duration, temporal trajectories, spatial propagation, or combinations of these properties. Information-theoretic approaches provide a potentially transferable framework: mutual information and channel capacity can quantify how reliably a signaling output discriminates among alternative inputs in the presence of noise, whereas complementary parameters such as temporal variance, phase relationships, propagation velocity, cross-correlation, and stimulus-response reproducibility can describe pathway-specific dynamics. Future cross-kingdom studies should therefore aim to establish standardized experimental designs in which defined stimuli and time-resolved signaling outputs are measured at single-cell or tissue resolution, allowing signal fidelity to be compared through common mathematical descriptors without assuming that the underlying molecular codes are identical.

A second unresolved issue is whether nanoplastic-induced perturbations in plant energy homeostasis converge on the SnRK1–TOR axis. SnRK1 and TOR constitute central and antagonistically coordinated regulators of plant energy status, metabolism, growth, and autophagy, making them plausible integration points for stress-associated changes in carbon availability and nutrient balance. Nanoplastics can alter ROS production, ion transport, nutrient homeostasis, and metabolic processes in plants, all of which could influence SnRK1–TOR signaling. However, direct evidence demonstrating the nanoplastic-dependent modification of SnRK1 or TOR kinase activity is currently lacking. Thus, SnRK1–TOR should not yet be considered an established primary target of nanoplastic stress. Future studies should combine time-resolved measurements of SnRK1/TOR activity and downstream phosphorylation events with genetic perturbation, pharmacological intervention, metabolomic profiling, and rescue or epistasis approaches to determine whether this axis is causally required for nanoplastic-induced metabolic and developmental responses.

A third major knowledge gap concerns how microbe-derived metabolites are perceived and molecularly decoded by plant cells. This limitation is particularly evident for reuterin. Although reuterin affects redox homeostasis and cellular responses in bacterial and animal systems, a specific receptor, transporter, or direct intracellular target has not been established in plants. Moreover, reuterin represents a chemically dynamic system containing 3-hydroxypropionaldehyde and related species, and evidence from mammalian cells indicates that its biological activity can involve glutathione depletion, selective protein oxidation, and electrophilic modification of protein cysteine residues. Consequently, receptor-independent chemical sensing or covalent modification should be considered alongside conventional ligand–receptor mechanisms. Determining whether reuterin or other microbial metabolites enter plant cells; determining their intracellular concentrations and chemical speciation; and identifying their protein, thiol, membrane, or metabolic targets using chemical proteomics, metabolomics, genetically encoded redox sensors, and targeted mutation screens will be essential for mechanistic validation of the metabolite-guided signal fidelity hypothesis.

More broadly, future studies should determine whether the preservation of signaling fidelity is causally responsible for increased stress tolerance rather than merely associated with it. This will require experiments that simultaneously quantify signaling dynamics and physiological outcomes, followed by interventions that selectively alter signaling variability, timing, or synchronization without simply changing mean pathway activity. Testing these predictions across phylogenetically distinct organisms under standardized perturbations will establish which elements of the proposed framework represent genuinely conserved principles and which reflect kingdom-specific adaptations. Such experiments will be critical for moving the signal–noise framework from a comparative conceptual model toward a quantitatively testable theory of biological stress responses.

## 11. Scope and Limitations

This integrative review prioritizes mechanistic compilation and conceptual unification over exhaustive quantitative aggregation. The evidence discussed spans multiple species, experimental platforms, and environmental stress paradigms across plants, animals, and microbial systems, which limits direct statistical comparison and meta-analytic inference. In addition, variability in exposure regimes, the temporal resolution of signaling measurements, and analytical methods constrain the precise quantification of signal variance and coherence across studies. Standardized live-cell and tissue imaging platforms, together with genetically encoded Ca^2+^, ROS, and redox biosensors, could improve temporal and spatial resolution while enabling quantitative comparisons of signal amplitude, frequency, variability, propagation, and coherence across experimental systems. Harmonized imaging protocols and analytical pipelines would further facilitate reproducible assessments of stochastic signaling perturbations. These limitations underscore the need for future standardized experimental frameworks and quantitative approaches to directly test the signal–noise predictions proposed here.

## 12. Conclusions

From a signal-fidelity perspective, future interventions should prioritize the preservation of biological information flow rather than solely mitigating molecular damage. Strategies that fortify metabolite-based redox buffering, stabilize ROS-Ca^2+^ signaling dynamics or maintain the integrity of central energy-sensing hubs such as TOR-related regulatory networks may help preserve systemic communication under sustained environmental stress. Although these mechanisms have been extensively characterized in plant systems, the underlying principle safeguarding spatiotemporal signaling coherence extends across biological kingdoms. Framing environmental toxicity as disruption of information fidelity rather than isolated cellular injury provides a unifying and actionable framework for engineering resilience in organisms operating within increasingly noisy and disrupted environments.

The signal-fidelity framework also has potential applications across agriculture, medicine, and ecosystem management. In agriculture, quantitative monitoring of ROS–Ca^2+^ dynamics and stress-induced signaling variability could support early detection of pollutant stress and guide interventions aimed at improving crop resilience, including antioxidant, metabolic, or microbiome-based strategies. In medicine, the framework may provide a complementary perspective for understanding how chronic environmental exposures interact with redox, metabolic, endocrine, and inflammatory signaling networks while emphasizing the need to distinguish mechanistic associations from clinically established effects. At the ecosystem level, measurements of plant, microbial, and animal signaling responses could help identify early molecular indicators of pollutant-induced ecological disruption and inform risk assessment and environmental management. In these applications, standardized biosensors, longitudinal measurements, and environmentally realistic exposure experiments will be essential for determining whether changes in signaling noise can serve as reliable predictors of physiological or ecological outcomes.

## Figures and Tables

**Figure 1:**
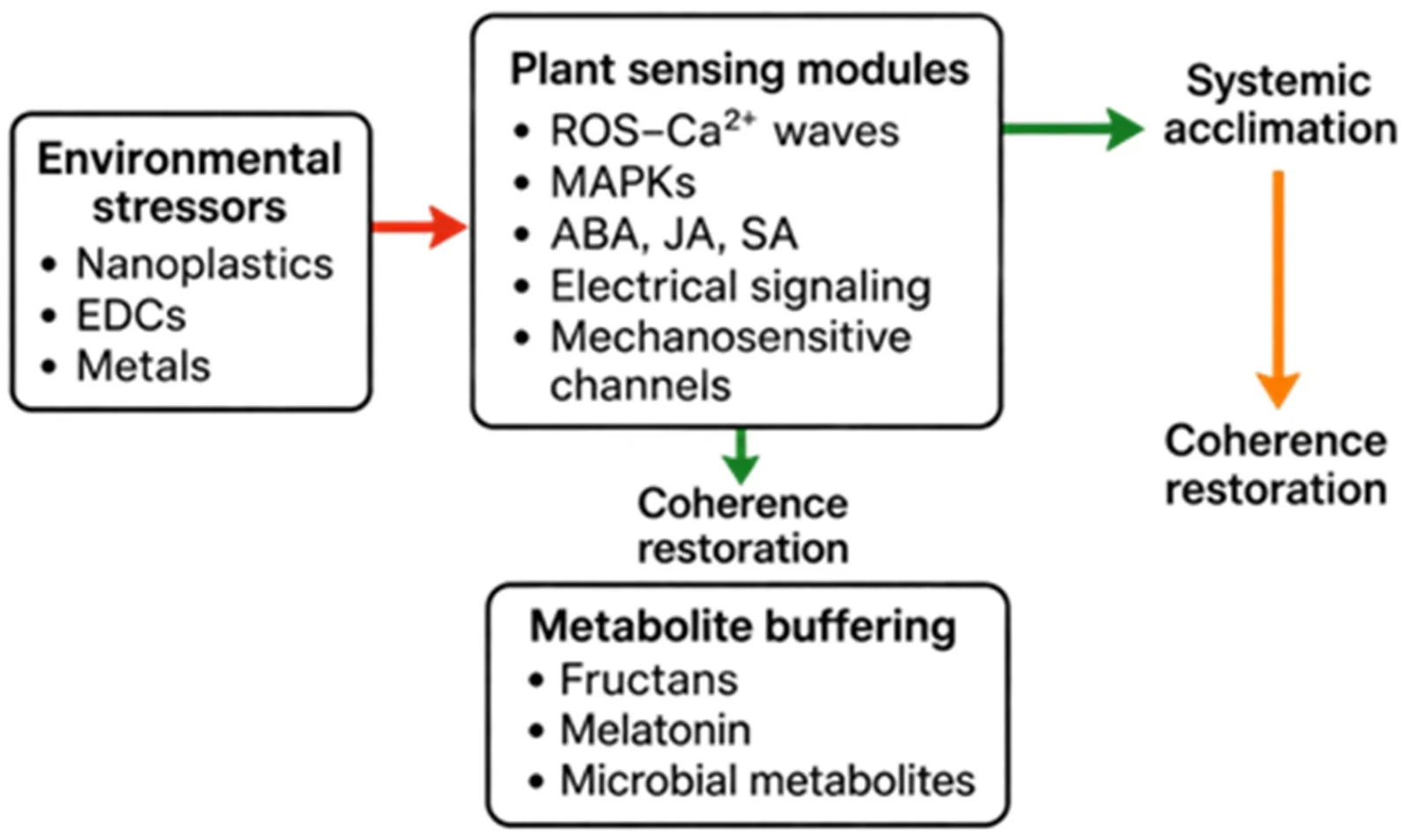
Signal fidelity and metabolite buffering in plant environmental stress sensing. Integrated phytohormonal networks, represented here by abscisic acid (ABA), jasmonic acid (JA), and salicylic acid (SA); EDCs, endocrine-disrupting chemicals; MAPKs, mitogen-activated protein kinases; and ROS, reactive oxygen species.

**Figure 2:**
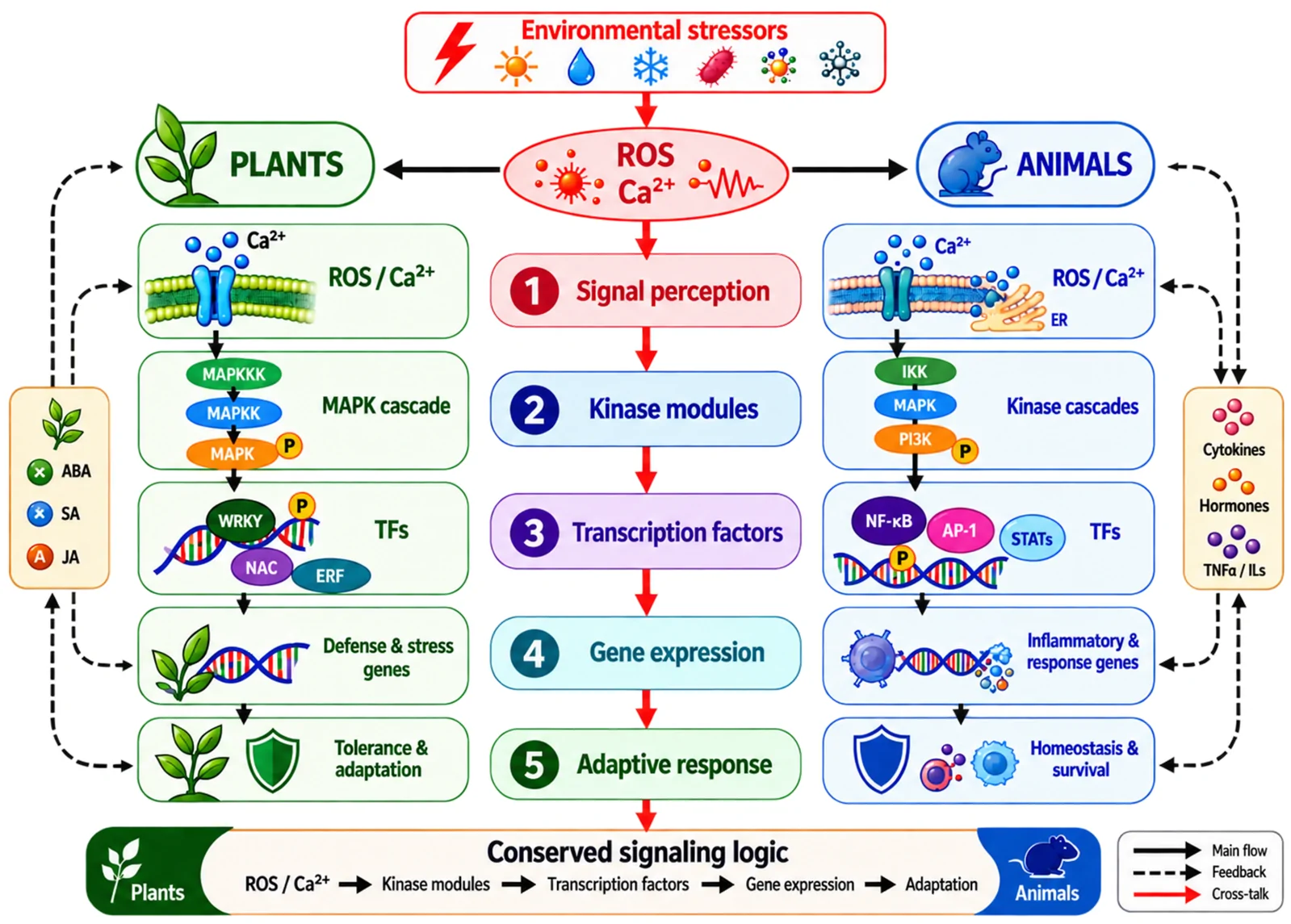
Mechanistic parallels in stress signaling between plants and animals. Comparative conceptual schematic illustrating functional and architectural parallels in stress-signaling networks across plants and animals. Both systems involve ROS–Ca^2+^ signaling, kinase cascades, transcriptional regulation, and hormonal or inflammatory feedback to coordinate stress responses. In plants, ROS–Ca^2+^ signaling is linked to MAPK cascades and stress-responsive transcription factor networks, including those of the WRKY, NAC, and ERF families, with ABA, jasmonic acid (JA), and salicylic acid (SA) representing selected components of the broader phytohormonal network. In animals, ROS–Ca^2+^ signaling interfaces with kinase modules, including IKK, MAPK, and PI3K, followed by transcriptional regulators such as NF-κB, AP-1, and STATs and downstream inflammatory, stress-response, and homeostatic programs. The correspondence between plant and animal modules represents functional and architectural analogy rather than direct genetic orthology or one-to-one molecular equivalence. The central ROS–Ca^2+^ node is depicted as a conceptual point of signal integration, whereas dashed connections represent regulatory feedback or crosstalk. Environmental stressors, including nanoplastics, metals, and endocrine-disrupting compounds (EDCs), are depicted as potential perturbations of these interconnected signaling networks. Green and blue elements distinguish plant and animal signaling architectures, respectively; red elements indicate environmental stress inputs and ROS–Ca^2+^-associated perturbations.

**Figure 3:**
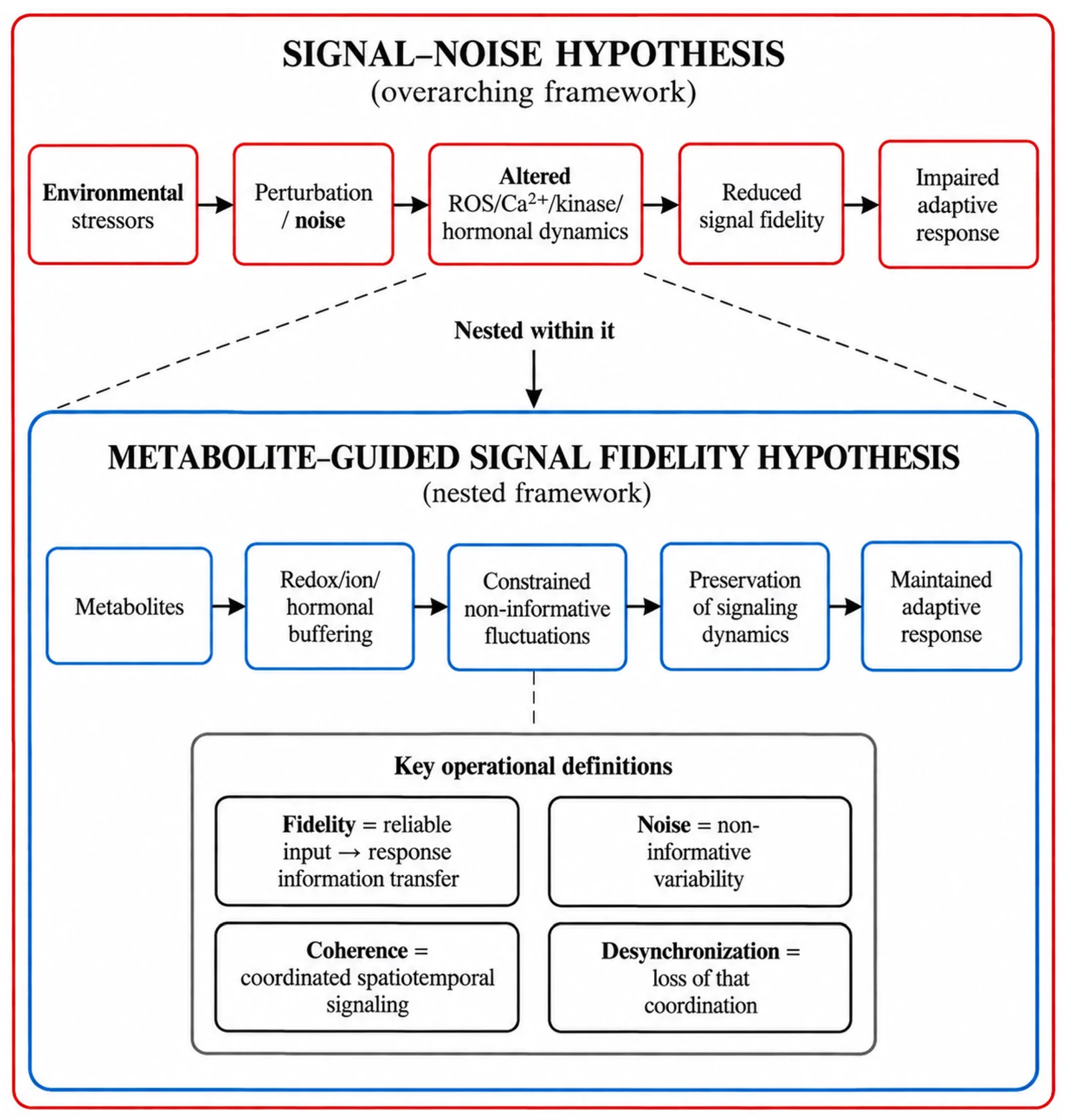
Conceptual schematic diagram illustrating the hierarchy of terminology adopted in this review. The overall framework is governed by the main signal–noise hypothesis, which addresses system-level information degradation under environmental stress. Nested within this main model is the metabolite-guided signal fidelity hypothesis, which proposes that specific endogenous and microbial metabolites act as stabilizers to preserve signaling reliability. Key operational definitions supporting these hypotheses, namely, Fidelity, Noise, Coherence, and Desynchronization, are defined at the base.

**Figure 4:**
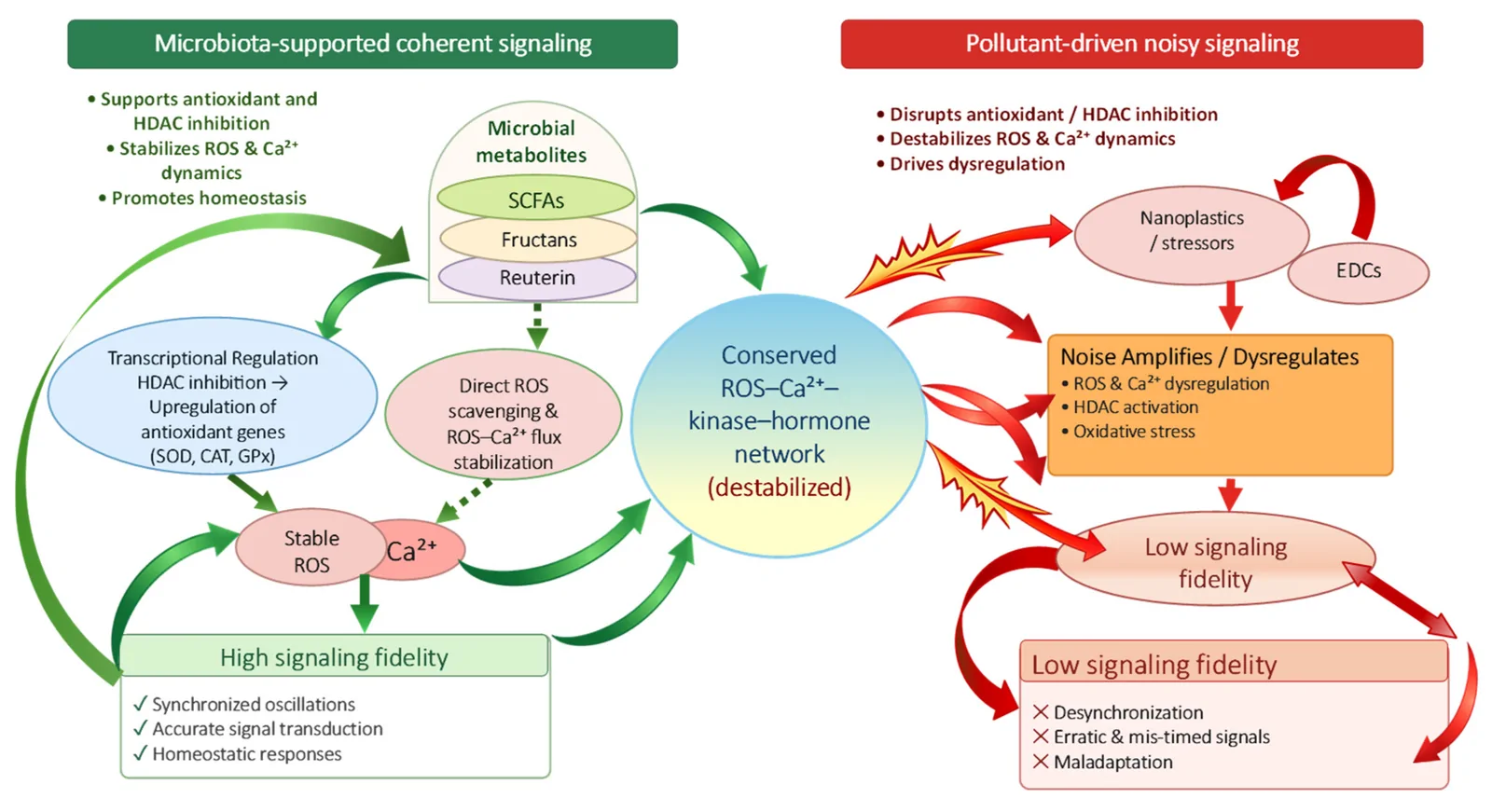
Metabolite-mediated stabilization of cross-kingdom stress signaling. The schematic places the ROS–Ca^2+^ hub at the center of stress-signal integration, linking redox and ionic dynamics with downstream kinase and hormonal networks. Microbial and plant metabolites, including short-chain fatty acids (SCFAs), fructans, and reuterin, stabilize signaling through two parallel routes: (i) transcriptional regulation, where SCFA-mediated HDAC inhibition upregulates antioxidant genes (SOD, CAT, GPx), and (ii) non-transcriptional buffering, where metabolites directly modulate ROS–Ca^2+^ fluxes. In contrast, nanoplastics, endocrine-disrupting compounds (EDCs), and other stressors perturb these interconnected networks, amplifying noise and driving maladaptive responses. Coherence (synchronized oscillations) and desynchronization (erratic signals) are shown here as conceptual systems-level properties of signaling fidelity and were not directly quantified in the present study.

**Figure 5:**
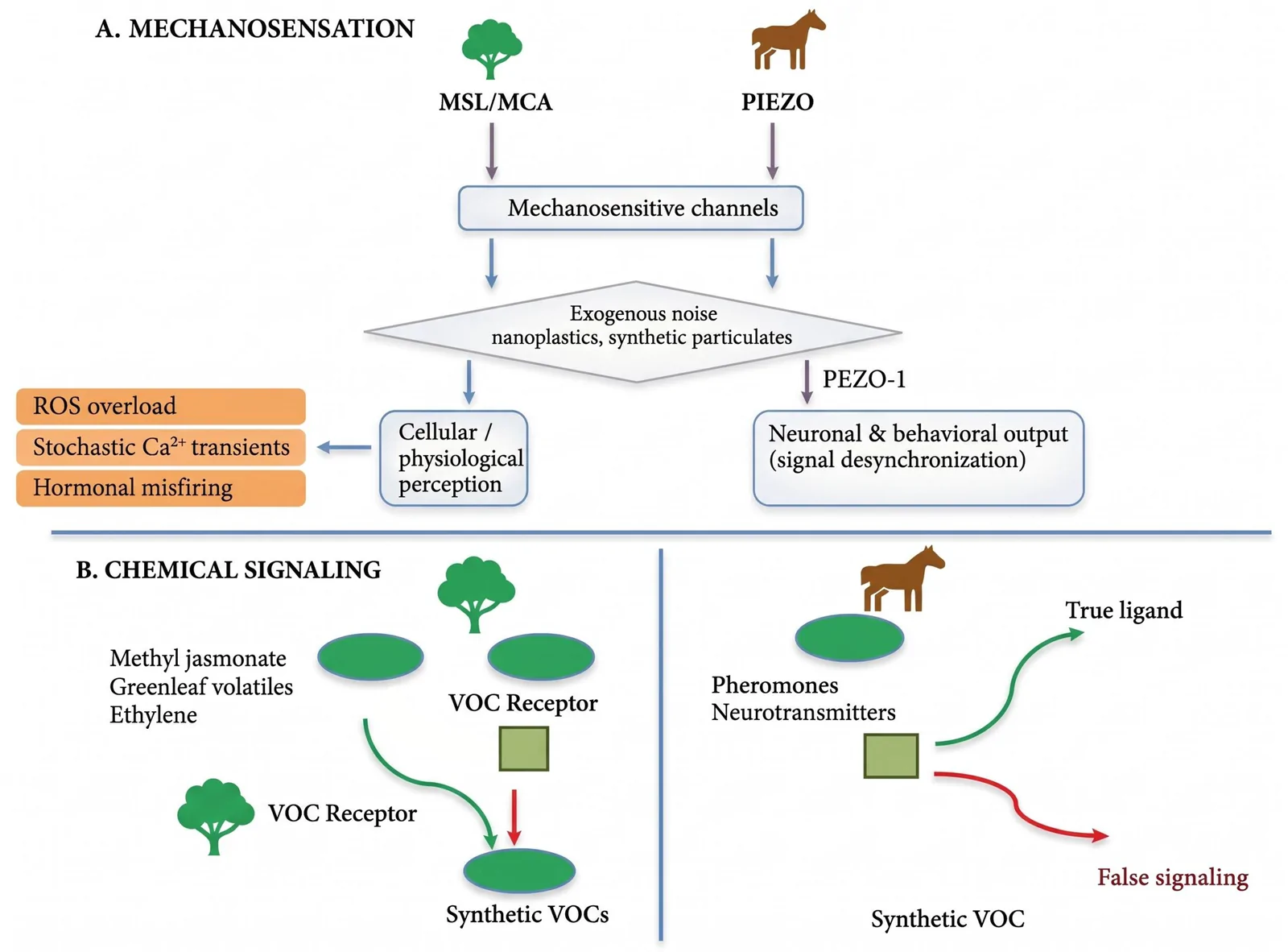
Cross-kingdom vulnerabilities in mechanosensory and chemical signaling. Nanoplastics and synthetic stressors can perturb conserved sensory architectures in plants and animals. (**A**) Representative mechanosensory systems, including plant MSL/MCA channels and animal Piezo-type channels, whose perturbation may alter downstream physiological and neuronal responses. The resulting effects may include altered reactive oxygen species (ROS) production, stochastic Ca^2+^ transients, and hormonal dysregulation. Experimental studies of mechanosensory perturbation and nanoplastic exposure provide evidence that altered channel activity can affect downstream physiological or behavioral outputs, thereby supporting the proposed link between sensory disruption and signaling desynchronization. (**B**) Chemical signaling fidelity is compromised when synthetic volatile organic compounds (VOCs) mimic or mask native ligands. In plants, this can affect receptors for methyl jasmonate, green leaf volatiles, and ethylene; in animals, it can interfere with pheromone and neurotransmitter recognition. These perturbations may generate false signaling events and reduce systemic coordination across biological systems.

**Figure 6:**
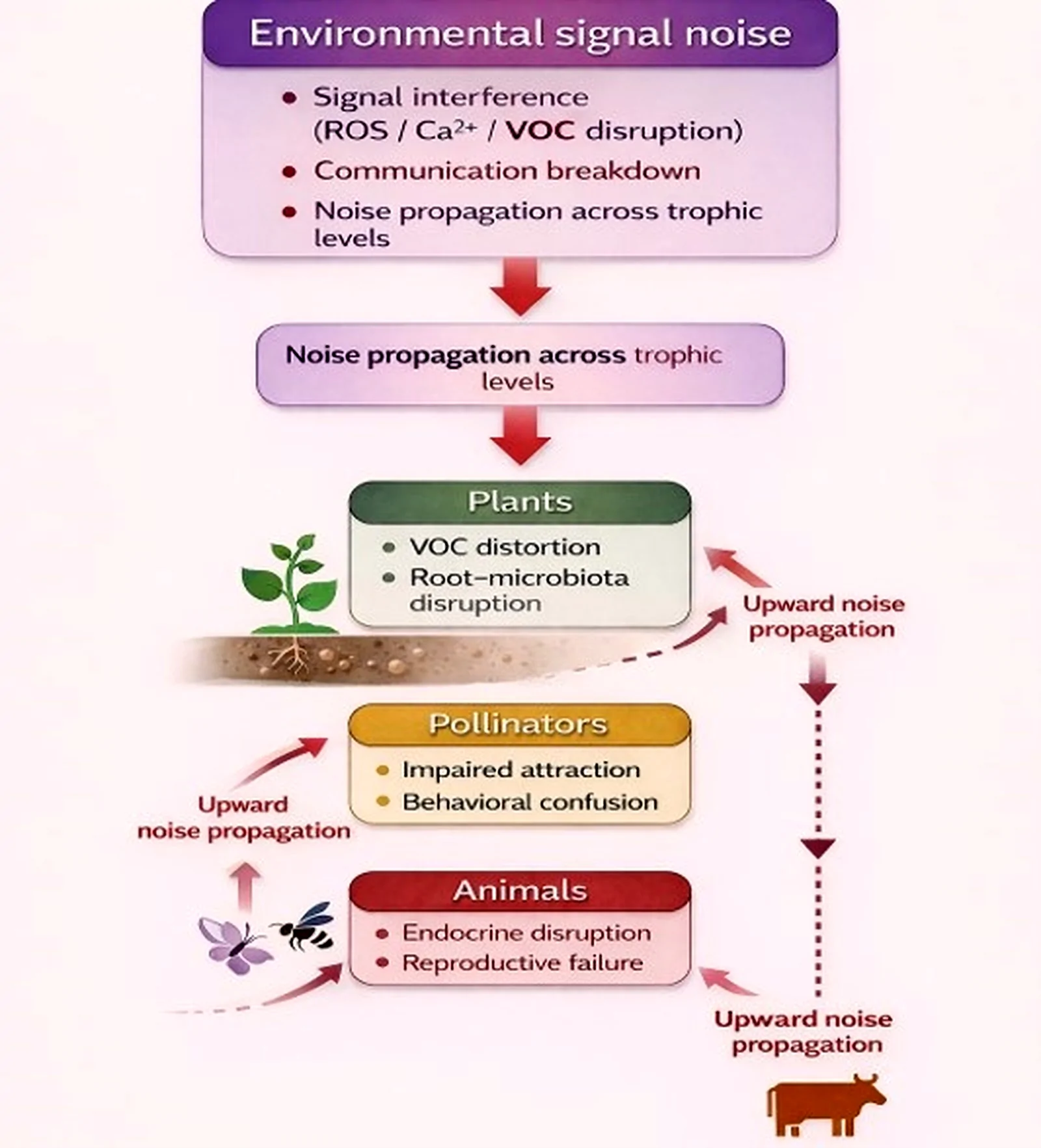
Ecosystem-level consequences of environmental signal noise. Conceptual diagram illustrating how disruption of ROS, Ca^2+^, and volatile organic compound (VOC) signaling can scale from molecular and organismal perturbations to altered ecological interactions. In plants, distorted volatile emissions may impair pollinator attraction and reproductive success, whereas nanoplastic-induced interference in the rhizosphere can disturb root–microbiota signaling. Pollinators may exhibit altered behavior and reduced foraging efficiency. In animals, endocrine disruption and reproductive effects can emerge as downstream consequences of impaired signaling coordination. Experimental studies have independently demonstrated pollutant-associated changes in plant VOC emissions, rhizosphere interactions, and animal sensory or endocrine responses, whereas the integrated cross-trophic propagation of signal noise shown here represents a conceptual framework that requires further direct validation.

**Figure 7:**
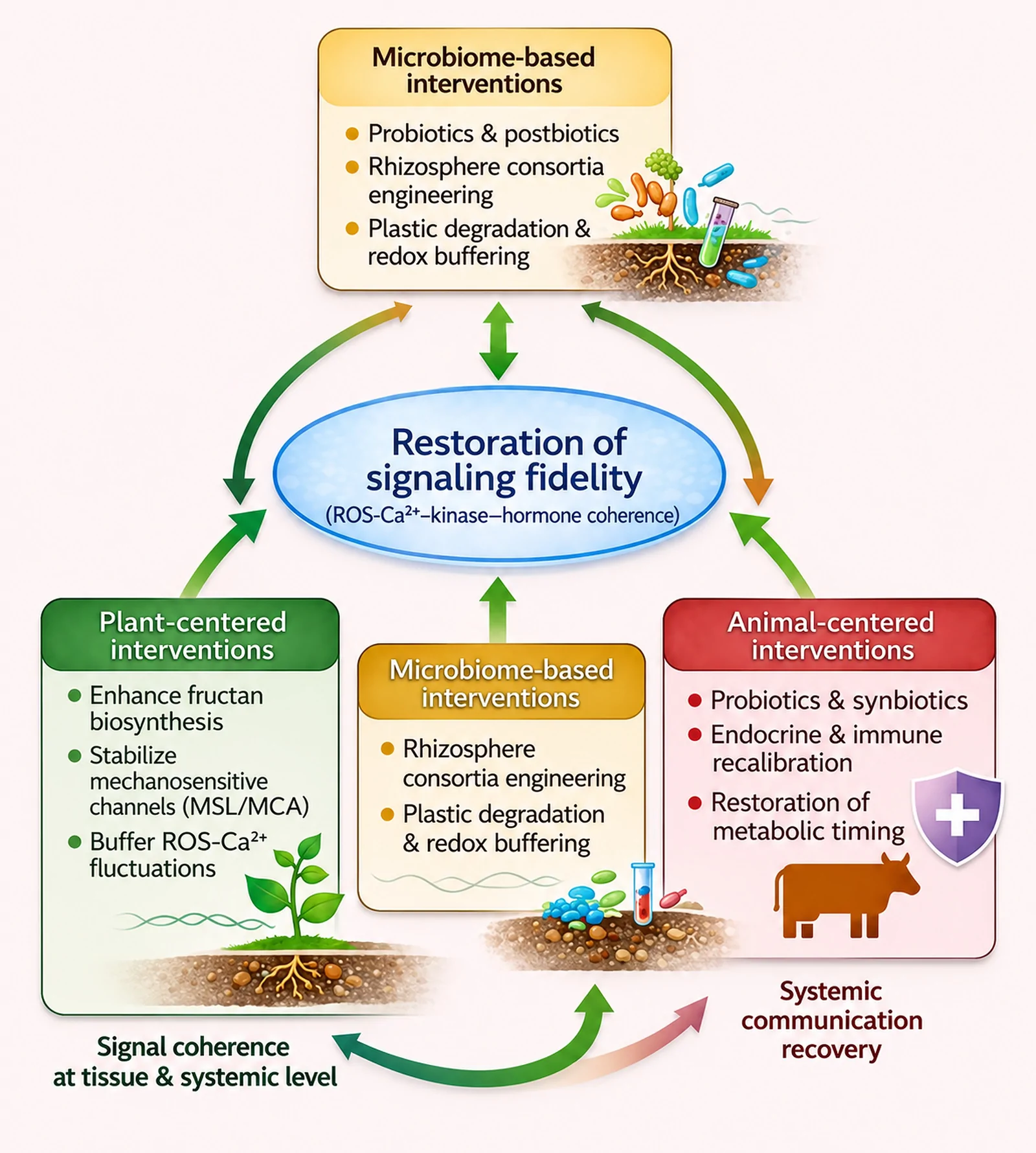
Translational strategies to restore signal fidelity under nanoscale pollutant stress. Conceptual framework illustrating plant-, microbiome- and animal-centered interventions aimed at counteracting the informational noise induced by nanoplastics. Plant-centered strategies include genetic enhancement of fructan biosynthesis, for example, through overexpression of fructan biosynthesis pathway genes, together with stabilization of mechanosensitive channels (MSL/MCA) and buffering of ROS–Ca^2+^ fluctuations. Microbiome-based interventions focus on probiotic and postbiotic support, rhizosphere consortia engineering, and microbial plastic degradation to restore redox-based communication. Animal-centered approaches involve symbiotic supplementation, endocrine and immune recalibration, and the restoration of metabolic timing. All strategies converge on the central goal of restoring ROS–Ca^2+^ kinase–hormone coordination and improving systemic signaling under environmental stress.

## Data Availability

No new data were created or analyzed in this study. Data sharing is not applicable to this article.

## Abbreviations

ABA	Abscisic acid
AKT	Protein kinase B
AMPK–mTOR	Adenosine 5′-monophosphate-activated protein kinase–mechanistic target of rapamycin
APX	Ascorbate peroxidase
BIN2	BRASSINOSTEROID-INSENSITIVE 2
BPA	Bisphenol A
Ca^2+^	Cytosolic calcium
CAND2	G-protein-coupled receptor CAND2
CAT	Catalase
CNGCs	Cyclic nucleotide-gated channels
CS-GT	Chitosan-gentamicin conjugate
EDCs	Endocrine-disrupting compounds
ER	Endoplasmic reticulum
ERF	Ethylene response factor
ETR	Ethylene response receptor
FSH	Follicle-stimulating hormone
GLR	Glutamate receptor-like channels
GPA1	G protein alpha subunit 1
GRP78	Glucose-regulated protein 78
H_2_O_2_	Hydrogen peroxide
HDAC	Histone deacetylase
HPCA1	Hydrogen peroxide-induced eroxide-induced ca^2+^ increases 1
HSF	Heat shock factor
HSP	Heat shock protein
HSP70	Heat shock protein 70
IRE1α	Inositol-requiring enzyme 1 alpha
JA	Jasmonic acid
JNK	c-Jun N-terminal kinase
LH	Luteinizing hormone
LHR	Luteinizing hormone receptor
LPS	Lipopolysaccharide
MAPK	Mitogen-activated protein kinase
MCAs	Mid1-complementing activity channel
MSLs	Mechanosensitive channel of small conductance-like channel
MT1/MT2	Melatonin receptor type 1/Melatonin receptor type 2
MYLK	Myosin light chain kinase
NF-κB	Nuclear factor kappa light chain enhancer of activated B cells
Nrf2/HO-1	Nuclear factor erythroid 2-related factor 2/heme oxygenase-1
PD	Parkinson’s disease
PET	Polyethylene terephthalate
PEZO-1	Piezo-type mechanosensitive ion channel component 1
PI3K	Phosphatidylinositol 3-kinase
PKA	Protein kinase A
PMTR1	Phytomelatonin Receptor 1
PYR/PYL	Pyrabactin resistance/Pyrabactin-resistance-like receptor group
RBOH/NADPH	Respiratory burst oxidase homolog/NADPH
ROS	Reactive oxygen species
SA	Salicylic acid
SCFAs	Short-chain fatty acids
SnRK1–TOR	Sucrose nonfermenting-1-related protein kinase 1-target of rapamycin
SOD	Superoxide dismutase
StAR	Steroidogenic acute regulatory protein
STAT6	Signal transducer and activator of transcription 6
TLR4	Toll-like receptor 4
TOR	Target of rapamycin
TPC1	Two-pore channel 1
ULC1	Unc-51-like autophagy activating kinase 1
UPR	Unfolded protein response
VOCs	Volatile organic compounds

## References

[1] Warda M., Tekin S., Gamal M., Khafaga N., Çelebi F., Tarantino G. (2025). Lipid rafts: Novel therapeutic targets for metabolic, neu-rodegenerative, oncological, and cardiovascular diseases. Lipids Health Dis..

[2] Angelovici R., Mittler R. (2024). Redox regulation of plant stress and development. Plant Cell Environ..

[3] Naz M., Afzal M.R., Raza M.A., Pandey S., Qi S., Dai Z., Du D. (2024). Calcium (Ca^2+^) signaling in plants: A plant stress perspective. S. Afr. J. Bot..

[4] Ravi B., Foyer C.H., Pandey G.K. (2023). The integration of reactive oxygen species (ROS) and calcium signalling in abiotic stress responses. Plant Cell Environ..

[5] da Silva C.J., Batista Fontes E.P., Modolo L.V. (2017). Salinity-induced accumulation of endogenous H2S and NO is associated with modulation of the antioxidant and redox defense systems in Nicotiana tabacum L. cv. Havana. Plant Sci..

[6] Waadt R., Seller C.A., Hsu P.-K., Takahashi Y., Munemasa S., Schroeder J.I. (2022). Plant hormone regulation of abiotic stress responses. Nat. Rev. Mol. Cell Biol..

[7] Guo F., Lv M., Zhang J., Li J. (2024). Crosstalk between Brassinosteroids and Other Phytohormones during Plant Development and Stress Adaptation. Plant Cell Physiol..

[8] Gilroy S., Białasek M., Suzuki N., Górecka M., Devireddy A.R., Karpiński S., Mittler R. (2016). ROS, Calcium, and Electric Signals: Key Mediators of Rapid Systemic Signaling in Plants. Plant Physiol..

[9] Toyota M., Spencer D., Sawai-Toyota S., Jiaqi W., Zhang T., Koo A.J., Howe G.A., Gilroy S. (2018). Glutamate triggers long-distance, calcium-based plant defense signaling. Science.

[10] Suda H., Toyota M. (2022). Integration of long-range signals in plants: A model for wound-induced Ca2+, electrical, ROS, and glutamate waves. Curr. Opin. Plant Biol..

[11] Yu A., Li P., Tang T., Wang J., Chen Y., Liu L. (2015). Roles of Hsp70s in Stress Responses of Microorganisms, Plants, and Animals. BioMed Res. Int..

[12] González-Bosch C., Zunszain P.A., Mann G.E. (2023). Control of Redox Homeostasis by Short-Chain Fatty Acids: Implications for the Prevention and Treatment of Breast Cancer. Pathogens.

[13] Tekin S., Sengul E., Yildirim S., Aksu E.H., Bolat İ., Çınar B., Shadidizaji A., Çelebi F., Warda M. (2024). Molecular insights into the antioxidative and anti-inflammatory effects of P-coumaric acid against bisphenol A-induced testicular injury: In vivo and in silico studies. Reprod. Toxicol..

[14] Adamczyk S., Chojak-Koźniewska J., Oleszczuk S., Michalski K., Velmala S., Zantis L.J., Bosker T., Zimny J., Adamczyk B., Sowa S. (2023). Polystyrene nanoparticles induce concerted response of plant defense mechanisms in plant cells. Sci. Rep..

[15] Li G., Qiu C., Zhang D., Lv M., Liao X., Li Q., Wang L. (2024). Effects of polystyrene nanoplastics (PSNPs) on the physiology of *Allium sativum* L.: Photosynthetic pigments, antioxidant enzymes, phytohormones, and nutritional quality. Environ. Exp. Bot..

[16] Cao X., Wang C., Luo X., Yue L., White J.C., Wang Z., Xing B. (2024). Nano- and Microplastics Increase the Occurrence of Bacterial Wilt in Tomato (*Solanum lycopersicum* L.). ACS Nano.

[17] Turgut Yiğit A., Yilmaz O., Uzilday B., Özgür Uzilday R., Türkan İ. (2020). Plant response to salinity: An analysis of ROS formation, signaling, and antioxidant defense. Turk. J. Bot..

[18] Secomandi E., De Gregorio M.A., Castro-Cegrí A., Lucini L. (2024). Single and combined effect of salinity, heat, cold, and drought in Arabidopsis at metabolomics and photosynthetic levels. bioRxiv.

[19] Hayashi T., Harada A., Sakai T., Takagi S. (2006). Ca2+ transient induced by extracellular changes in osmotic pressure in Arabidopsis leaves: Differential involvement of cell wall–plasma membrane adhesion. Plant Cell Environ..

[20] Stephan A.B., Kunz H.-H., Yang E., Schroeder J.I. (2016). Rapid hyperosmotic-induced Ca 2+ responses in Arabidopsis thaliana exhibit sensory potentiation and involvement of plastidial KEA transporters. Proc. Natl. Acad. Sci. USA.

[21] da-Silva C.J., Mollica D.C.F., Vicente M.H., Peres L.E.P., Modolo L.V. (2018). NO, hydrogen sulfide does not come first during tomato response to high salinity. Nitric Oxide.

[22] You J., Chan Z. (2015). ROS Regulation During Abiotic Stress Responses in Crop Plants. Front. Plant Sci..

[23] Gao Y., Ma X., Zhang Z., Wang Y. (2026). Adversity Response: Multiple Defense Mechanisms Mediated by Plant Hormones in Reg-ulating ROS Networks. J. Plant Growth Regul..

[24] Lebeau P.F., Platko K., Byun J.H., Austin R.C. (2021). Calcium as a reliable marker for the quantitative assessment of endoplasmic reticulum stress in live cells. J. Biol. Chem..

[25] Moccia F., Fiorio Pla A., Lim D., Lodola F., Gerbino A. (2023). Intracellular Ca2+ signalling: Unexpected new roles for the usual suspect. Front Physiol..

[26] Maroto M., Monk N.A.M. (2009). Cellular Oscillatory Mechanisms.

[27] Li J., Cui J., Li X., Zhu D., Chen Z., Huang X., Wang Y., Wu Q., Tian Y. (2025). TMBIM-2 orchestrates systemic mitochondrial stress response via facilitating Ca2+ oscillations. J. Cell Biol..

[28] Bharatula V., Broach J.R. (2018). The Nutrient Stress Response in Yeast. Stress Response Mechanisms in Fungi.

[29] Aranguiz K., Horianopoulos L.C., Elkin L., Abá K.S., Jordahl D., Overmyer K.A., Wrobel R.L., Coon J.J., Shiu S.-H., Rokas A. (2025). Machine learning reveals genes impacting oxidative stress resistance across yeasts. Nat. Commun..

[30] Huang H., Huang C., Guo L., Zeng C., Ye H. (2019). Profiles of calreticulin and Ca2+ concentration under low temperature and salinity stress in the mud crab, *Scylla paramamosain*. PLoS ONE.

[31] Roychowdhury P., Aftabuddin M., Pati M. (2024). A Review on The Impact of Thermal Stress on Fish Biochemistry. Aquat. Sci. Eng..

[32] Chaudhry Z.L., Gamal M., Ferhati I., Warda M., Ahmed B.Y. (2022). ER Stress in COVID-19 and Parkinson’s Disease: In Vitro and In Silico Evidences. Brain Sci..

[33] Bej E., Cesare P., Volpe A.R., d’Angelo M., Castelli V. (2024). Oxidative Stress and Neurodegeneration: Insights and Therapeutic Strategies for Parkinson’s Disease. Neurol. Int..

[34] Jia F., Han J. (2024). COVID-19 related neurological manifestations in Parkinson’s disease: Has ferroptosis been a suspect?. Cell Death Discov..

[35] Mao G., Meng X., Liu Y., Zheng Z., Chen Z., Zhang S. (2011). Phosphorylation of a WRKY Transcription Factor by Two Patho-gen-Responsive MAPKs Drives Phytoalexin Biosynthesis in Arabidopsis. Plant Cell.

[36] Sheng W., Ji G., Zhang L. (2023). Immunomodulatory effects of inulin and its intestinal metabolites. Front. Immunol..

[37] Dasmahapatra A.K., Chatterjee J., Tchounwou P.B. (2025). A systematic review of the effects of nanoplastics on fish. Front. Toxicol..

[38] Lin R.-H., Chen H.-T., Lee I.-T., Vo T.-T.-T., Wang Y.-L. (2025). Microplastics and Nanoplastics in Health Concerning Cellular Toxicity Mechanisms, Exposure Pathways, and Global Mitigation Strategies. Life.

[39] Jiang X., Qiu H., Li X., Guo W., Peijnenburg W.J.G.M., He E. (2025). Species-Specific Foliar Absorption and Translocation of Nanoplastics in Leafy Vegetables Revealed through Isotopic, Physiological, and Transcriptomic Analyses. ACS Nano.

[40] Sengul E., Yildirim S., Cinar İ., Tekin S., Dag Y., Bolat M., Gok M., Warda M. (2024). Mitigation of Acute Hepatotoxicity Induced by Cadmium Through Morin: Modulation of Oxidative and Pro-apoptotic Endoplasmic Reticulum Stress and Inflammatory Responses in Rats. Biol. Trace Elem. Res..

[41] Lamontagne F., Paz-Trejo C., Zamorano Cuervo N., Grandvaux N. (2024). Redox signaling in cell fate: Beyond damage. Biochim. Et. Biophys. Acta (BBA)-Mol. Cell Res..

[42] Ekner-Grzyb A., Duka A., Grzyb T., Lopes I., Chmielowska-Bąk J. (2022). Plants oxidative response to nanoplastic. Front. Plant Sci..

[43] Sun L., Wang X., Zhao H., Wang Z., Zhao Y., Huang H., Yang R., Wang S., Zhao W. (2023). Micro/nanoplastics: A potential threat to crops. Veg. Res..

[44] Azeem I., Adeel M., Ahmad M.A., Shakoor N., Zain M., Yousef N., Yinghai Z., Azeem K., Zhou P., White J.C. (2022). Microplastic and Nanoplastic Interactions with Plant Species: Trends, Meta-Analysis, and Perspectives. Environ. Sci. Technol. Lett..

[45] Mansoor S., Ali A., Kour N., Bornhorst J., AlHarbi K., Rinklebe J., Abd El Moneim D., Ahmad P., Chung Y.S. (2023). Heavy Metal Induced Oxidative Stress Mitigation and ROS Scavenging in Plants. Plants.

[46] Khalid F., Rasheed Y., Ashraf H., Asif K., Maqsood M.F., Shahbaz M., Zulfiqar U., Farhat F., Nawaz S., Ahmad M. (2025). Nanoparti-cle-Mediated Phytohormone Interplay: Advancing Plant Resilience to Abiotic Stresses. J. Crop Health.

[47] Prylutska S.V., Tkachenko T.A., Tkachenko V.V., Yemets A.I. (2024). The Role of Aquaporins and Carbon Nanomaterials in Abiotic Stress in Plants. Cytol. Genet.

[48] Zhou J., Yu Y., Luan Y., Dai W. (2022). The Formation of Protein Corona by Nanoplastics and Horseradish Peroxidase. Nanomaterials.

[49] Vilanova O., Martinez-Serra A., Monopoli M.P., Franzese G. (2025). Characterizing the hard and soft nanoparticle-protein corona with multilayer adsorption. Front. Nanotechnol..

[50] Shi R., Xia P., Yang X., Zhang X., Liu S., Shen R., Tan H., Deng Y., Zhang Y. (2026). Health impacts of micro- and nanoplastics: Key influencing factors, limitations, and future perspectives. Arch. Toxicol..

[51] Mei W., Chen W., Wang Y., Liu Z., Dong Y., Zhang G., Deng H., Liu X., Lu X., Wang F. (2023). Exogenous Kinetin Modulates ROS Homeostasis to Affect Heat Tolerance in Rice Seedlings. Int. J. Mol. Sci..

[52] Yang Y., Luo J., Feng J., Hu H., Xiao J., Ding C., Nangia V., Liu Y. (2026). Root‐Derived Trans‐ Zeatin‐Type Cytokinins Increase Cold Tolerance in Rice Seedlings by Regulating Energy Metabolism. Plant Cell Environ..

[53] Chen T., Hayes M., Liu Z., Isenegger D., Mason J., Spangenberg G. (2024). Modified fructan accumulation through overexpression of wheat fructan biosynthesis pathway fusion genes Ta1SST:Ta6SFT. BMC Plant Biol..

[54] Liu H., Zhang Z., Li J., Liu W., Warda M., Cui B., Abd El-Aty A.M. (2022). Oligosaccharides derived from Lycium barbarum ameliorate glycolipid metabolism and modulate the gut microbiota community and the faecal metabolites in a type 2 diabetes mouse model: Metabolomic bioinformatic analysis. Food Funct..

[55] Li J., Wang T., Yang X., Duan Y., Mori H. (2025). Systematic analysis of the antibacterial mechanisms of reuterin using the *E. coli* Keio collection. mBio.

[56] Engevik M.A., Danhof H.A., Shrestha R., Chang-Graham A.L., Hyser J.M., Haag A.M., Mohammad M.A., Britton R.A., Versalovic J., Sorg J.A. (2020). Reuterin disrupts Clostridioides difficile metabolism and pathogenicity through reactive oxygen species generation. Gut Microbes.

[57] Purnawita W., Rahayu W.P., Lioe H.N., Nurjanah S., Wahyudi S.T. (2024). Potential molecular mechanism of reuterin on the inhibition of *Aspergillus flavus* conidial germination: An in silico study. J. Food Sci..

[58] Xu Y., Ding X., Wang Y., Li D., Xie L., Liang S., Zhang Y., Li W., Fu A., Zhan X. (2022). Bacterial Metabolite Reuterin Attenuated LPS-Induced Oxidative Stress and Inflammation Response in HD11 Macrophages. Antioxidants.

[59] Frolova M.S., Suvorova I.A., Iablokov S.N., Petrov S.N., Rodionov D.A. (2022). Genomic reconstruction of short-chain fatty acid pro-duction by the human gut microbiota. Front. Mol. Biosci..

[60] Duan H., Wang L., Huangfu M., Li H. (2023). The impact of microbiota-derived short-chain fatty acids on macrophage activities in disease: Mechanisms and therapeutic potentials. Biomed. Pharmacother..

[61] Nshanian M., Gruber J.J., Geller B.S., Chleilat F., Lancaster S.M., White S.M., Alexandrova L., Camarillo J.M., Kelleher N.L., Zhao Y. (2025). Short-chain fatty acid metabolites propionate and butyrate are unique epigenetic regulatory elements linking diet, metabolism and gene expression. Nat. Metab..

[62] Niu X., Hu C., Chen S., Wen J., Liu X., Yong Y., Yu Z., Ma X., Li C., Warda M. (2023). Chi-tosan-gentamicin conjugate attenuates heat stress-induced intestinal barrier injury via the TLR4/STAT6/MYLK signaling pathway: In vitro and in vivo studies. Carbohydr. Polym..

[63] Monteiro K.K.A.C., Shiroma M.E., Damous L.L., de Jesus Simões M., dos Santos Simões R., Cipolla-Neto S., Baracat E.C., Soares-Jr. J.M. (2024). An-tioxidant Actions of Melatonin: A Systematic Review of Animal Studies. Antioxidants.

[64] Aijaz I., Kwon E.-H., Lee I.-J., Khan A.L. (2025). Melatonin microbe interactions in plant rhizosphere. iScience.

[65] Khan M., Ali S., Manghwar H., Saqib S., Ullah F., Ayaz A., Zaman W. (2022). Melatonin Function and Crosstalk with Other Phyto-hormones under Normal and Stressful Conditions. Genes.

[66] Wei J., Li D., Zhang J., Shan C., Rengel Z., Song Z., Chen Q. (2018). Phytomelatonin receptor PMTR 1‐mediated signaling regulates stomatal closure in Arabidopsis thaliana. J. Pineal Res..

[67] Chen Q., Dai H., Kou J., Chen Y., Liu X., Huang W., Wang X., Chen Q., Liao H., Pu X. (2025). Interaction between HPCA1 and melatonin signaling components modulates stomatal closure and drought tolerance in Arabidopsis thaliana. Plant Physiol. Biochem..

[68] Horta L.P., Braga M.R., Lemos-Filho J.P., Modolo L.V. (2014). Organ-coordinated response of early post-germination mahogany seed-lings to drought. Tree Physiol..

[69] Rajput V.D., Harish, Singh R.K., Verma K.K., Sharma L., Quiroz-Figueroa F.R., Meena M., Gour V.S., Minkina T., Sushkova S. (2021). Recent Developments in Enzymatic Antioxidant Defence Mechanism in Plants with Special Reference to Abiotic Stress. Biology.

[70] Mishra N., Jiang C., Chen L., Paul A., Chatterjee A., Shen G. (2023). Achieving abiotic stress tolerance in plants through antioxidative defense mechanisms. Front. Plant Sci..

[71] Benkeblia N. (2022). Insights on Fructans and Resistance of Plants to Drought Stress. Front. Sustain. Food Syst..

[72] Sun K., Pan Y.-T., Jiang H.-J., Xu J.-Y., Ma C.-Y., Zhou J., Liu Y., Shabala S., Zhang W., Dai C.-C. (2024). Root endophyte-mediated alteration in plant H2O2 homeostasis regulates symbiosis outcome and reshapes the rhizosphere microbiota. J. Exp. Bot..

[73] Pavlů J., Novák J., Koukalová V., Luklová M., Brzobohatý B., Černý M. (2018). Cytokinin at the Crossroads of Abiotic Stress Signalling Pathways. Int. J. Mol. Sci..

[74] Bulgakov V.P., Avramenko T.V. (2020). Linking Brassinosteroid and ABA Signaling in the Context of Stress Acclimation. Int. J. Mol. Sci..

[75] Sun D., Tong W., Han S., Wu M., Li P., Li Y., Liang Y. (2025). Effects of Dietary Supplementation with Lactobacillus reuteri Postbiotics on Growth Performance, Intestinal Flora Structure and Plasma Metabolome of Weaned Piglets. Animals.

[76] Selimkhanov J., Taylor B., Yao J., Pilko A., Albeck J., Hoffmann A., Tsimring L., Wollman R. (2014). Accurate information transmission through dynamic biochemical signaling networks. Science.

[77] Tyc H.J., Kłodnicka K., Teresińska B., Karpiński R., Flieger J., Baj J. (2025). Micro- and Nanoplastics as Disruptors of the Endocrine System—A Review of the Threats and Consequences Associated with Plastic Exposure. Int. J. Mol. Sci..

[78] Bergman T.J., Beehner J.C. (2023). Information Ecology: An integrative framework for studying animal behavior. Trends Ecol. Evol..

[79] Han T., Wang L., Jiang Q., Wang M., Wang C., Wang M., Zhao J., Tian S., Liu C., Yang Y. (2024). Extracellular adenosine triphosphate (eATP) alleviates phytotoxicity of nanoplastics in maize (*Zea mays* L.) plants. Environ. Exp. Bot..

[80] Smit J.A.H., Vooijs R., Lindenburg P., Baugh A.T., Halfwerk W. (2024). Noise and light pollution elicit endocrine responses in urban but not forest frogs. Horm. Behav..

[81] Yoshimura K., Iida K., Iida H. (2021). MCAs in Arabidopsis are Ca2+-permeable mechanosensitive channels inherently sensitive to membrane tension. Nat. Commun..

[82] Zhang Z., Ye F., Xiong T., Chen J., Cao J., Chen Y., Liu S. (2023). Origin, evolution and diversification of plant mechanosensitive channel of small conductance-like (MSL) proteins. BMC Plant Biol..

[83] Kurusu T., Kuchitsu K., Tada Y. (2015). Plant signaling networks involving Ca2+ and Rboh/Nox-mediated ROS production under salinity stress. Front. Plant Sci..

[84] Qu M., Zhao X., Wang Q., Xu X., Chen H., Wang Y. (2024). PIEZO mediates a protective mechanism for nematode Caenorhabditis elegans in response to nanoplastics caused dopaminergic neurotoxicity at environmentally relevant concentrations. Ecoto-Xicol Environ. Saf..

[85] Brosset A., Blande J.D. (2022). Volatile-mediated plant–plant interactions: Volatile organic compounds as modulators of receiver plant defence, growth, and reproduction. J. Exp. Bot..

[86] Liu S., Feng S., Zhang Y., Zheng Y., Jiang R., Ouyang G. (2025). Impact of microplastics on plant biogenic volatile organic compounds emission: A preliminary study. J. Hazard Mater..

[87] Luo J., Yu W., Xiao Y., Zhang Y., Peng F. (2022). Overexpression of PpSnRK1α in Tomato Increased Autophagy Activity under Low Nutrient Stress. Int. J. Mol. Sci..

[88] Rawat S.S., Laxmi A. (2024). Sugar signals pedal the cell cycle!. Front. Plant Sci..

[89] Feng L., Li X., Zheng X.-A., Zheng Z., Liu Q.-R., Liu C., Zhu Q.-L., Shen W., Yang C., Li H. (2025). SnRK1 and TOR: Central regulators of autophagy in plant energy stress responses. Abiotech.

[90] Guo Y., Liu L., Fan Y., Du S., Chen Y., Duan Y., Han R., Xu S., Wen G., Zhou W. (2024). Polyethylene terephthalate nanoplastics affect potassium accumulation in foxtail millet (*Setaria italica*) seedlings. BMC Plant Biol..

[91] Jin H., Yan M., Pan C., Liu Z., Sha X., Jiang C., Li L., Pan M., Li D., Han X. (2022). Chronic exposure to polystyrene microplastics induced male reproductive toxicity and decreased testosterone levels via the LH-mediated LHR/cAMP/PKA/StAR pathway. Part Fibre Toxicol..

[92] Liu Y., Li X., Xiong Y. (2024). Chronic Polystyrene Microplastic Exposure Reduces Testosterone Levels in Mice through Mitochondrial Oxidative Stress and BAX/BCL2-Mediated Apoptosis. Toxics.

[93] Lin M., Hua R., Ma J., Zhou Y., Li P., Xu X., Yu Z., Quan S. (2021). Bisphenol A promotes autophagy in ovarian granulosa cells by inducing AMPK/mTOR/ULK1 signalling pathway. Environ. Int..

[94] Lin Y.-F., Aarts M.G.M. (2012). The molecular mechanism of zinc and cadmium stress response in plants. Cell. Mol. Life Sci..

[95] Zhang M., Dong C. (2025). Molecular Mechanisms of Cadmium Stress Resistance in Vegetable Crops. Int. J. Mol. Sci..

[96] Wang W., Zhou C., Ma Z., Zeng L., Wang H., Cheng X., Zhang C., Xue Y., Yuan Y., Li J. (2024). Co-exposure to polystyrene nanoplastics and triclosan induces synergistic cytotoxicity in human KGN granulosa cells by promoting reactive oxygen species accumulation. Ecotoxicol. Environ. Saf..

[97] Geng Y., Liu Z., Hu R., Huang Y., Li F., Ma W., Wu X., Dong H., Song K., Xu X. (2023). Toxicity of microplastics and nanoplastics: Invisible killers of female fertility and offspring health. Front. Physiol..

[98] Zhang Z., Meng J., Tian J., Li N., Chen Z., Yun X., Song D., Li F., Duan S., Zhang L. (2024). Reproductive and developmental implications of micro- and nanoplastic internalization: Recent advances and perspectives. Ecotoxicol. Environ. Saf..

[99] Li C.-C., Jhou S.-M., Li Y.-C., Ciou J.-W., Lin Y.-Y., Hung S.-C., Chang J.-H., Chang J.-C., Sun D.-S., Chou M.-L. (2022). Exposure to low levels of photocatalytic TiO2 nanoparticles enhances seed germination and seedling growth of amaranth and cru-ciferous vegetables. Sci. Rep..

[100] Xu T., Niu J., Jiang Z. (2022). Sensing Mechanisms: Calcium Signaling Mediated Abiotic Stress in Plants. Front. Plant Sci..

[101] Yadav M., Singh A. (2025). Interplay of Calcium Sensors with ROS: Unravelling the Crosstalk in Plant Defense Response. J. Plant Growth Regul..

[102] Shah S., Ilyas M., Li R., Yang J., Yang F.-L. (2023). Microplastics and Nanoplastics Effects on Plant–Pollinator Interaction and Polli-nation Biology. Environ. Sci. Technol..

[103] Benson N.U., Agboola O.D., Fred-Ahmadu O.H., De-la-Torre G.E., Oluwalana A., Williams A.B. (2022). Micro(nano)plastics Prevalence, Food Web Interactions, and Toxicity Assessment in Aquatic Organisms: A Review. Front. Mar. Sci..

[104] Corsi I., Bellingeri A., Bergami E. (2023). Progress in selecting marine bioindicators for nanoplastics ecological risk assessment. Ecol. Indic..

[105] Crisp P.A., Ganguly D., Eichten S.R., Borevitz J.O., Pogson B.J. (2016). Reconsidering plant memory: Intersections between stress recovery, RNA turnover, and epigenetics. Sci. Adv..

[106] Dhaka V., Singh S., Rao R., Garg S., Samuel J., Khan N.A., Ramamurthy P.C., Singh J. (2025). Statistical optimization of process variables for improved poly(ethylene terephthalate) plastic degradation by a rhizospheric bacterial consortium. Sci. Rep..

[107] Hao Y., Min J., Ju S., Zeng X., Xu J., Li J., Wang H., Shaheen S.M., Bolan N., Rinklebe J. (2024). Possible hazards from biodegradation of soil plastic mulch: Increases in microplastics and CO2 emissions. J. Hazard Mater..

[108] Zhao S.-Y., Meng Y.-L., Yang Z.-H., Li B.L., Li Y.-Y., Han H., Liu L., Duan P.-F., Chen Z.-J. (2025). Rhizosphere microbiome metagenomics in PGPR-mediated alleviation of combined stress from polypropylene microplastics and Cd in hybrid Pennisetum. Front. Microbiol..

[109] Bazeli J., Banikazemi Z., Hamblin M.R., Sharafati Chaleshtori R. (2023). Could probiotics protect against human toxicity caused by polystyrene nanoplastics and microplastics?. Front. Nutr..

